# Supplementary material for: Structure-Guided Development of a Potent BioA Inhibitor Validates Biotin Synthesis Inhibition as a Therapeutic Strategy for Tuberculosis
Source: bioRxiv. 2025 Sep 24:2025.09.24.678246. Preprint. [Version 1] doi: 10.1101/2025.09.24.678246 (PMC12485673; doi:10.1101/2025.09.24.678246)
Supplement: Supplement 1 [file media-1.docx]

##### Structure-Guided Development of a Potent BioA Inhibitor Validates Biotin Synthesis Inhibition as a Therapeutic Strategy for Tuberculosis

Qiang Liu^a†^, Joshua B. Wallach^b†^, Yahani P. Jayasinghe^c^, Mark R. Sullivan^d^, Julianna Proietto^e^, Suyapa Rodriguez^e^, Sang Vo^a,f^, Helena I. M. Boshoff^g^, Ziyi Jia^f^, Lev Ostrer^f^, Kritee Mehdiratta^d^, Rui Shi^a^, Véronique Dartois^e^, Anthony D. Baughn^f^, Eric J. Rubin^d^, Donald R. Ronning^c^, Matthew D. Zimmerman*^e^, Dirk Schnappinger*^b^, and Courtney C. Aldrich*^a^

^†^contributed equally

Table of Contents

[General materials and methods. 3](#_Toc208957969)

[Supplemental Scheme S1. Synthesis of C21 and C48 4](#_Toc208957970)

[Synthesis of C21. 4](#_Toc208957971)

[Synthesis of Piperazine 10. 5](#_Toc208957972)

[Synthesis of C48. 5](#_Toc208957973)

[Supplemental Table S1. Reported biotin biosynthesis inhibitors in literature^2^ 7](#_Toc208957974)

[Supplemental Table S2. Crystallographic Data of C48 8](#_Toc208957975)

[Supplemental Figure S1. Cocrystal of C48/BioA 10](#_Toc208957976)

[Supplemental Table S4. MIC screening of C48 against a panel of pathogens and strains 14](#_Toc208957977)

[Structure Alignment of MtBioA and ESKAPE BioA (Figure S2, Table S5 and S6) 15](#_Toc208957978)

[Supplemental Table S7. C48 Resistant Mutants and AA change 20](#_Toc208957979)

[Supplemental Figure S3. Spleen efficacy of C48 in conventional mice model 21](#_Toc208957980)

[Chemistry 22](#_Toc208957981)

[^1^H and ^13^C NMR Spectra 22](#_Toc208957982)

[HSQC of C48 25](#_Toc208957983)

[^19^F NMR of C48 26](#_Toc208957984)

[HPLC Trace of C21 27](#_Toc208957985)

[HPLC Trace of C48 27](#_Toc208957986)

[HRMS of C21 28](#_Toc208957987)

[HRMS of compound 10 29](#_Toc208957988)

[HRMS of C48 30](#_Toc208957989)

**General materials and methods.**

Chemicals and solvents were purchased from Alfa Aesar, Oakwood chemicals, Chem-Impex International, Acros Organics, TCI America and Sigma-Aldrich, and were directly used as received. An anhydrous solvent dispensing system using two packed columns of molecular sieves were used for drying DMF, while two packed columns of neutral alumina were applied to dry CH_2_Cl_2_, and the solvents were dispensed under nitrogen gas (N_2_). Anhydrous grade dioxane was purchased from Sigma-Aldrich. EtOAc and hexanes were purchased from Fisher Scientific. All reactions were performed in oven-dried (150 °C) glassware under an inert atmosphere of dry nitrogen gas (N_2_). TLC analyses were carried out on TLC silica gel plates 60F_254_ purchased from Sigma-Aldrich and were visualized by UV light lamp. Purification by flash chromatography was performed using a medium-pressure flash chromatography system (Buchi) equipped with flash column silica cartridges with the indicated solvent system. Analytical reversed-phase HPLC purity was performed on a Waters XSelect 5 μm C18 150 × 4.6 mm column operating at 1 mL/min with detection at 254 nm employing a linear gradient from 5% to 95% MeCN (0.1% FA) in H_2_O (0.1% FA) for 10 min. ^1^H, ^13^C and ^19^F spectrums were acquired on 400 or 600 MHz NMR spectrometers (Agilent Scientific Instruments). Proton chemical shifts are recorded in ppm by an internal standard of residual dimethyl sulfoxide (2.50), methanol (3.31) and chloroform (7.26); carbon chemical shifts are recorded in ppm from an internal standard of residual dimethyl sulfoxide (39.5), methanol (49.1), or chloroform (77.2). Proton chemical data are reported as follows: chemical shift, multiplicity (s = singlet, d = doublet, dt = doublet of triplets, t = triplet, q = quartet, m = multiplet, ap = apparent, br = broad, ovlp = overlapping), coupling constant (s), integration. Melting points of final compounds were determined by Thomas Hoover capillary melting point apparatus. High-resolution mass spectra were obtained on an LTQ Orbitrap Velos (Thermo Scientific, Waltham, MA). All final compounds were determined to be > 95% purity by analytical reverse-phase HPLC. All animal studies were ethically reviewed and carried out in compliance with the Institutional Animal Care and Use Committee of Hackensack Meridian Health.

**Supplemental Scheme S1.** Synthesis of **C21** and **C48**

# *Synthesis of* ***C21***.

To a solution of carboxylic acid **6** (114 mg, 0.50 mmol, 1.0 equiv) and known amine **7**^1^ (130 mg, 0.60 mmol, 1.2 equiv) in CH_2_Cl_2_ (10 mL) EDCI (116 mg, 0.75 mmol, 1.5 equiv) and DMAP (6 mg, 0.050 mmol, 0.10 equiv) were added and the reaction was stirred for 16 h at 23 °C, quenched with addition of water (25 mL) and extracted by CH_2_Cl_2_ (3 × 20 mL). The combined organic layers were washed with brine, dried over Na_2_SO_4_ and concentrated under reduced pressure. Purification by flash chromatography on silica gel (1:3 hexanes/EtOAc) afforded the product **C21** (155 mg, 85%) as a white solid; *R*_f_ = 0.4 (1:3 hexanes/EtOAc); ^1^H NMR (400 MHz, CDCl_3_) δ 7.68 (dd, *J* = 6.5, 2.1 Hz, 1H), 7.65 (d, *J* = 8.6 Hz, 1H), 7.39 (ddd, *J* = 8.4, 4.6, 2.1 Hz, 1H), 7.19 (t, *J* = 8.3 Hz, 1H), 6.88 (dd, *J* = 8.7, 2.2 Hz, 1H), 6.83 (s, 1H), 4.12–3.51 (br, 4H), 3.42 (s, 4H), 3.11–2.98 (m, 2H), 2.72–2.60 (m, 2H); ^13^C NMR (101 MHz, CDCl_3_) δ 205.2, 168.2, 160.2 (d, *J*_C-F_ = 253.0 Hz), 158.0, 155.4, 133.0, 132.8 (d, *J*_C-F_ = 4.2 Hz), 129.1, 128.4 (d, *J*_C-F_ = 7.8 Hz), 125.4, 116.9 (d, *J*_C-F_ = 22.9 Hz), 115.1, 110.9, 109.8 (d, *J*_C-F_ = 21.8 Hz), 48.1, 36.5, 26.1; HRMS (ESI+) *m/z* calcd for C_20_H_19_BrFN_2_O_2_ [M+H]^+^ 417.0608, found 417.0604 (error 1 ppm). Melting point: 168 ℃.

# *Synthesis of Piperazine* ***10***.

To a 100 mL flask charged with N_2_, commercially available **8** (500 mg, 3.0 mmol, 1.0 equiv), amine **9** (670 mg, 3.6 mmol, 1.2 equiv) and DMSO (15 mL) were added, followed by the addition of DIPEA (780 mg, 6 mmol, 2.0 equiv). The reaction was stirred at 110 °C for 3 h, quenched with water (20 mL) and extracted by EtOAc (3 × 30 mL). The combined organic layers were washed with brine, dried with Na_2_SO_4_ and concentrated under reduced pressure. Purification by flash chromatography on silica gel (1:1 hexanes/EtOAc) afforded the Buchwald coupling product **10** (789 mg, 83%) as a grey solid. *R*_f_ = 0.3 (1:1 hexanes/EtOAc); ^1^H NMR (400 MHz, CDCl_3_) δ 8.61 (s, 1H), 6.53 (s, 1H), 3.72 (t, *J* = 5.2 Hz, 4H), 3.55 (t, *J* = 5.4 Hz, 4H), 3.16–2.91 (m, 2H), 2.78–2.49 (m, 2H), 1.49 (s, 9H); ^13^C NMR (101 MHz, CDCl_3_) δ 203.64, 164.71, 161.63, 154.93, 146.66, 123.99, 101.63, 81.49, 44.87, 36.42, 28.60, 25.80; HRMS (ESI+) *m/z* calcd for C_17_H_24_N_3_O_3_ [M+H]^+^ 318.1818, found 318.1820 (error 0.6 ppm).

# *Synthesis of* ***C48***.

To a solution of compound **10** (500 mg, 1.5 mmol, 1.0 equiv) in CH_2_Cl_2_ (10 mL) was added 4 M HCl in dioxane (5 mL) at 0 °C and the solution was stirred at 23 °C for 4 h. The solvent was removed and the crude product amine was dried under vacuum and used for the next step without further purification.

The above deprotection product was dissolved in CH_2_Cl_2_ (15 mL) and subsequently acid **6** (501 mg, 2.3 mmol, 1.5 equiv), EDCI (100 mg, 2.3 mmol, 1.5 equiv) and DMAP (18 mg, 0.15 mmol, 0.10 equiv) were added and the mixture was stirred at 23 °C for 8 h. The reaction was quenched with water (25 mL) and extracted by CH_2_Cl_2_ (3 × 20 mL). The combined organic layers were washed with brine, dried with Na_2_SO_4_ and concentrated under reduced pressure. Purification by flash chromatography on silica gel (EtOAc) afforded the product **C48** (538 mg, 86%) as a white solid, *R*_f_ = 0.3 (1:2 hexanes/EtOAc); ^1^H NMR (400 MHz, CDCl_3_) δ 8.54 (s, 1H), 7.90–7.57 (m, 1H), 7.41–7.31 (m, 1H), 7.15 (t, *J* = 8.3 Hz, 1H), 6.55 (s, 1H), 3.99–3.44 (m, 8H), 2.99 (t, *J* = 6.2 Hz, 2H), 2.69–2.30 (m, 2H); ^13^C NMR (101 MHz, CDCl_3_) δ 203.4, 168.2, 164.7, 161.2, 160.0 (d, *J*_C-F_ = 252.9 Hz), 146.23, 132.97, 132.76 (d, *J* = 4.1 Hz), 128.29 (d, *J* = 7.7 Hz), 124.29, 116.81 (d, *J* = 22.9 Hz), 109.65 (d, *J* = 21.4 Hz), 101.92, 44.9, 36.2, 25.7; ^19^F NMR (376 MHz, CDCl_3_) *δ* –103.7 (q, *J* = 6.2 Hz); HRMS (ESI+) *m/z* calcd for C_19_H_18_BrFN_3_O_2_ [M+H]^+^ 418.0566, found 418.0553 (error 0.7 ppm). Melting point: 182 ℃.

# **Supplemental Table S1.** Reported biotin biosynthesis inhibitors in literature^2^

| Structure | Target Enzyme | Activity |
| --- | --- | --- |
|  | BioA | IC_50_ of 155 nM against *Mt*BioA and MIC of 26 µM againt *Mtb*^1^ |
|  | BioA | ﻿Ki: 10.4 µM against *Mt*BioA^3^ |
|  | BioA | ﻿IC_50_: 250 nM against *E. coli* BioA and MIC: 8 µg/mL against *E. coli*^4^ |
|  | BioA | ﻿Ki: 12 µM against *Mt*BioA^5^ |
|  | BioB | ﻿Ki: 1 µM against *Mt*BioB and MIC: 0.6 µg/mL against *Mtb*^6^ |
|  | BioB | ﻿MIC: 8 µg/mL against *E. coli*  0.5 µg/mL against *B. subtilis*  0.2 µg/mL against *M. avium*^7^ |
|  | BioC | ﻿0.1 µM reduced 60% *B. cereus*  BioC activity, complete inhibition of BioC at 10 µM^8^ |
|  | BioD | ﻿Ki: 11 mM against *E. coli* BioD^9^ |
|  | BioF | *K*i = 7 µM against *Bacillus sphaericus* BioF^10^ |

# **Supplemental Table S2.** Crystallographic Data of C**48**

|  | **BioA_C48 (PDB: 9D7M)** |
| --- | --- |
| Wavelength | 0.97 |
| Resolution range | 62.83 - 1.97 (2.04 - 1.97) |
| Space group | P 21 21 21 |
| Unit cell |  |
| a, b, c (Å) | 63.1373 66.2907 204.841 |
| α, β, γ (°) | 90 90 90 |
| Total reflections | 614033 (16106) |
| Unique reflections | 61199 (5713) |
| Multiplicity | 11.3 (5.5) |
| Completeness (%) | 96.22 (80.60) |
| Mean I/sigma(I) | 37.85 (8.29) |
| Wilson B-factor | 20.37 |
| R-merge | 0.053 (0.16) |
| R-meas | 0.055 (0.17) |
| R-pim | 0.016 (0.07) |
| CC1/2 | 0.999 (0.97) |
| CC* | 1 (0.99) |
| Reflections used in refinement | 59446 (4904) |
| Reflections used for R-free | 1846 (112) |
| R-work | 0.1536 (0.19) |
| R-free | 0.1913 (0.24) |
| CC(work) | 0.962 (0.909) |
| CC(free) | 0.947 (0.869) |
| Number of non-hydrogen atoms | 7496 |
| macromolecules | 6488 |
| ligands | 82 |
| solvent | 926 |
| Protein residues | 860 |
| RMS(bonds) | 0.004 |
| RMS(angles) | 0.84 |
| Ramachandran favored (%) | 97.2 |
| Ramachandran allowed (%) | 2.1 |
| Ramachandran outliers (%) | 0.7 |
| Rotamer outliers (%) | 0 |
| Clashscore | 3.45 |
| Average B-factor | 21.68 |
| macromolecules | 20.37 |
| ligands | 16.95 |
| solvent | 31.31 |
| Statistics for the highest-resolution shell are shown in parentheses. | |

# **Supplemental Figure S1.** Cocrystal of **C48/**BioA

**
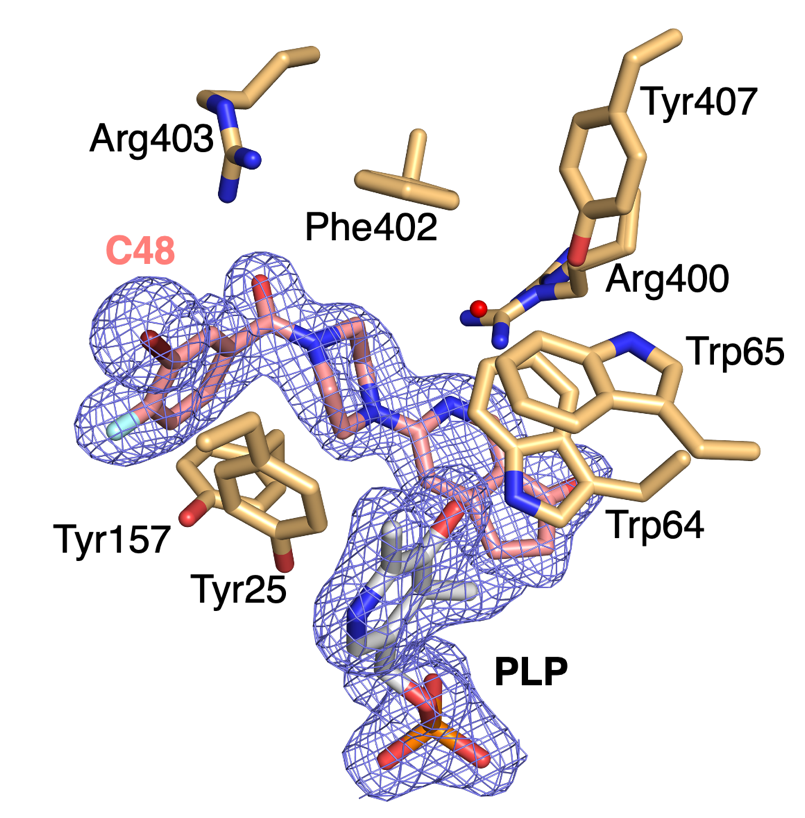
**

**Supplemental Figure S1.** The calculated 2Fo-Fc omit map highlighting **C48** and PLP in the BioA active site. The map is contoured at 1σ in purple. The carbon atoms of **C48** are pink, the carbon atoms of the BioA residues are tan, and the carbon atoms of PLP are gray. The nitrogen, oxygen, phosphorous, fluorine, and bromine atoms are blue, red, orange, cyan, and maroon, respectively.

**Supplemental Table S3.** Bacterial strains and cell lines used in this study

| Category | Genotype | Reference/Source |
| --- | --- | --- |
| *M.* tuberculosis (M. *tb*) | Wild-type (H37Rv) | ^11^ |
| *H37Rv ΔbioA TetON-1 (BioA-OE)* | BioA-Overexpress | ^12^ |
| *H37Rv ΔbioA TetON-5 (BioA-UE)* | BioA-Underexpress | ^12^ |
| *M. tb* HN878 | DS | ^13-15^ |
| *M. tb* Erdman | DS | ^13-15^ |
| *M. tb* *CDC1551* | DS | ^13-15^ |
| *M. tb* K03b00DS | DS | ^13-15^ |
| *M. tb* K04b00DS | DS | ^13-15^ |
| *M. tb* K05b00DS | DS | ^13-15^ |
| *M. tb* K07b00DS | DS | ^13-15^ |
| *M. tb* K08b00DS | DS | ^13-15^ |
| *M. tb* K09b00DS | DS | ^13-15^ |
| *M. tb* K10b00DS | DS | ^13-15^ |
| *M. tb* K11b00DS | DS | ^13-15^ |
| *M. tb* K12b00DS | DS | ^13-15^ |
| *M. tb* K13b00DS | DS | ^13-15^ |
| *M. tb* K14b00DS | DS | ^13-15^ |
| *M. tb* K15b00DS | DS | ^13-15^ |
| *M. tb* K16b00DS | DS | ^13-15^ |
| *M. tb* K17b00DS | DS | ^13-15^ |
| *M. tb* K35b00DS | DS | ^13-15^ |
| *M. tb* NIH_G13 | DS | ^13-15^ |
| *M. tb* NIH_G1DS | DS | ^13-15^ |
| *M. tb* NIH_G36 | DS | ^13-15^ |
| *M. tb* NIH_GA36 | DS | ^13-15^ |
| *M. tb* NIH_SA1 | DS | ^13-15^ |
| *M. tb* NIH_SA3 | DS | ^13-15^ |
| *M. tb* NIH_SA39 | DS | ^13-15^ |
| *M. tb* NIH_SA23 | DS | ^13-15^ |
| *M. tb* NIH_SA27 | DS | ^13-15^ |
| *M. tb* NIH_SA43 | DS | ^13-15^ |
| *M. tb* NIH_SA125 | DS | ^13-15^ |
| *M. tb* NIH_G12 | DS | ^13-15^ |
| CRC clinical strain K5072429 | DS | ^13-15^ |
| *M. tb* NIH_G9R | MCs | ^13-15^ |
| *M. tb* NIH_G10R | HRESRb | ^13-15^ |
| *M. tb* NIH_G11R | ECs | ^13-15^ |
| *M. tb* NIH_G16R | SCs | ^13-15^ |
| *M. tb* NIH_G17R | HEtCs | ^13-15^ |
| *M. tb* NIH_G21R | HRECsRb | ^13-15^ |
| *M. tb* NIH_G22R | SCs | ^13-15^ |
| *M. tb* NIH_G76MR | HROML | ^13-15^ |
| *M. tb* NIH1B314 | HEtCsOThLZ | ^13-15^ |
| *M. tb* 001K113 | HRSKPCpAkPtOMLRbZ | ^13-15^ |
| *M. tb* 28K111 | HRESKPCpAkPtCsOMLRbZLz | ^13-15^ |
| *M. tb* OK116 | HRESOMLPtCsRb | ^13-15^ |
| *M. tb* K37b00XR | HEKOPRM | ^13-15^ |
| *M. tb* CRC clinical strain 00202293 | HRESKPEtML | ^13-15^ |
| *M. tb* CRC clinical strain 01291696 | HRS | ^13-15^ |
| *M. tb* 026K111 | HRESKPCpAkPtCsOMLRbZLz | ^13-15^ |
| *M. tb* 053K113 | HRSKPCpAkPtOMLRbZLz | ^13-15^ |
| *M. tb* K21b00MR | HRES | ^13-15^ |
| *M. tb* K22b00MR | HRERb | ^13-15^ |
| *M. tb* K25b00MR | HREZRbTh | ^13-15^ |
| *M. tb* K26b00MR | HREZRb | ^13-15^ |
| *M. tb* CRC clinical MDR strain 01291696 | HRS | ^13-15^ |
| *M. tb* K18b01MR | HRERb | ^13-15^ |
| *M. tb* K29b00MR | HRSPO | ^13-15^ |
| *M. tb* NIH_G269DR | HRERb | ^13-15^ |
| *M. tb* NIH_G367DR | HROM | ^13-15^ |
| *M. tb* K20b00MR | HREZSKP | ^13-15^ |
| *M. tb* K32b00MR | HREKP | ^13-15^ |
| *M. tb* K33b00MR | HREZSKPTh | ^13-15^ |
| *M. tb* NIH_G5MR | HREKO | ^13-15^ |
| *M. tb* NIHB188 | HRZThRbLOM | ^13-15^ |
| *M. tb* Kb019 | HREPKOTh | ^13-15^ |
| *M*. bovis 0AF2122 | DS | ^13-15^ |
| *M. africanum* | DS | ^13-15^ |
| *M. abscessus* (ATCC19977) | Reference strain | ^16^ |
| *M. abscessus* T35 | Clinical strain | ^16^ |
| *M. abscessus* T37 | Clinical strain | ^16^ |
| *M. abscessus* T38 | Clinical strain | ^16^ |
| *M. abscessus* T49 | Clinical strain | ^16^ |
| *M. abscessus* BWH-B | Clinical strain | ^16^ |
| *M. abscessus* BWH-D | Clinical strain | ^16^ |
| *E. coli* C0244 | Gram-negative | IIDR clinical isolate collection |
| *E. coli* BW | Gram-negative | ^2^ |
| *A. baumannii* ATCC 17978 | Gram-negative | https://www.atcc.org/products/17978 |
| *K. pneumoniae* ATCC 43816 | Gram-negative | ^2^ |
| *P. aeruginosa* PA01 | Gram-negative | ^2^ |
| *E. faecium* ATCC 19434 | Gram-negative | https://genomes.atcc.org/genomes/b6  890c9e9268452d |
| *S. aureus* | Gram-positive | ^17^ |
| HepG2 | Mammalian | from ATCC |
| HT-29 | Mammalian | from ATCC |
| DS = Drug Susceptible Strains. Letter codes represent drug(s) to which that strain is resistant. Ak = Amikacin, Cp = Capreomycin, Cs = D-Cycloserine, E = Ethambutol, Et = Ethionamide, H = Isoniazid, K = Kanamycin, L = Levofloxacin, Lz = Linezolid, M = Moxifloxacin, O = Ofloxacin, P = *p*-Aminosalicylic acid, R = Rifampicin, Rb = Rifabutin, S = Streptomycin, Pt = Prothionamide, Z = Pyrazinamide, Th = Thiacetazone. | | |

# **Supplemental Table S4.** MIC screening of **C48** against a panel of pathogens and strains

| Strains | Type | **C48** MIC (µM)^b^ | Linezolid MIC (µM)^b^ |
| --- | --- | --- | --- |
| *M. tb* Erdman | DS^a^ | 0.024 | 1.6 |
| *M. tb* K08b00DS | DS^a^ | < 0.024 | 1.56 |
| *M. tb* K15b00DS | DS^a^ | < 0.024 | 1.56 |
| *M. tb* K16b00DS | DS^a^ | < 0.024 | 1.56 |
| *M. tb* K17b00DS | DS^a^ | < 0.024 | 1.56 |
| *M. tb* K35b00DS | DS^a^ | < 0.024 | 1.2 |
| *M. tb* CRC clinical strain K5072429 | DS^a^ | < 0.024 | 1.2 |
| *M. tb* NIH_G12 | DS^a^ | < 0.024 | 2.3 |
| *M. tb* NIH_G1DS | DS^a^ | < 0.024 | 2.3 |
| *M. tb* NIH_GA36 | DS^a^ | < 0.024 | 1.6 |
| *M. tb* NIH_SA3 | DS^a^ | < 0.024 | ND^c^ |
| *M. tb* NIH_SA23 | DS^a^ | < 0.024 | ND^c^ |
| *M. tb* NIH_SA27 | DS^a^ | < 0.024 | ND^c^ |
| *M. tb* NIH_SA39 | DS^a^ | < 0.024 | ND^c^ |
| *M. tb* NIH_SA43 | DS^a^ | < 0.024 | ND^c^ |
| *M. tb* NIH_SA125 | DS^a^ | < 0.024 | ND^c^ |
| *M. abscessus* (ATCC19977) | Reference strain | 0.5^d^ | ND^c^ |
| *M. abscessus* T35 | Clinical strain | 0.5^d^ | ND^c^ |
| *M. abscessus* T37 | Clinical strain | 1^d^ | ND^c^ |
| *M. abscessus* T38 | Clinical strain | 1^d^ | ND^c^ |
| *M. abscessus* T49 | Clinical strain | 0.5^d^ | ND^c^ |
| *M. abscessus* BWH-B | Clinical strain | 0.5^d^ | ND^c^ |
| *M. abscessus* BWH-D | Clinical strain | 0.5^d^ | ND^c^ |
| *E. coli* C0244 | Gram-negative | inactive | ND^c^ |
| *E. coli* BW | Gram-negative | inactive | ND^c^ |
| *A. baumannii* ATCC 17978 | Gram-negative | inactive | ND^c^ |
| *K. pneumoniae* ATCC 43816 | Gram-negative | inactive | ND^c^ |
| *P. aeruginosa* PA01 | Gram-negative | inactive | ND^c^ |
| *E. faecium* ATCC 19434 | Gram-negative | inactive | ND^c^ |
| *S. aureus* | Gram-positive | inactive | ND^c^ |
| Type: DS^a^ = drug-sensitive Mtb clinical isolates. MIC^b^ = minimum inhibitory concentrations that resulted in complete growth inhibition. ND^c^ = Not determined. MIC^d^ = minimum inhibitory concentrations that resulted in 50% growth inhibition. | | | |

|  |
| --- |

# **Structure Alignment of *Mt*BioA and ESKAPE BioA (Figure S2, Table S5 and S6)**

**>NP_216084.1 *Mycobacterium tuberculosis***

MAAATGGLTPEQIIAVDGAHLWHPYSSIGREAVSPVVAVAAHGAWLTLIRDGQPIEVLDAMSSWWTAIHGHGHPALDQALTTQLRVMNHVMFGGLTHEPAARLAKLLVDITPAGLDTVFFSDSGSVSVEVAAKMALQYWRGRGLPGKRRLMTWRGGYHGDTFLAMSICDPHGGMHSLWTDVLAAQVFAPQVPRDYDPAYSAAFEAQLAQHAGELAAVVVEPVVQGAGGMRFHDPRYLHDLRDICRRYEVLLIFDEIATGFGRTGALFAADHAGVSPDIMCVGKALTGGYLSLAATLCTADVAHTISAGAAGALMHGPTFMANPLACAVSVASVELLLGQDWRTRITELAAGLTAGLDTARALPAVTDVRVCGAIGVIECDRPVDLAVATPAALDRGVWLRPFRNLVYAMPPYICTPAEITQITSAMVEVARLVGSLP

**>WP_000131427.1 *Acinetobacter baumannii***

MTDNFDLEHIWHPYTSMTQPLPTFKVKRAYGATIELDDGRTLIDGMSSWWCAIHGYNHPELNQAVTDQLQNMSHIMFGGLTHDPAIELGKILLKITPPSLDKIFYADSGSVAVEVALKMAVQFWTAQGQPQKTNFITTRSGYHGDTWNAMSVCDPVTGMHQIFGTSLPNRLFVAAPQTKFHEEWNQEDIAELEQAIQQNHENLAALIIEPIVQGAGGMRFYHPEYLRQAKALCEKYHLLLIFDEIATGFGRTGKLFAWEHAQVEPDIMCLGKGLTGGYMTLSATLTTKHVAETISRGEAGVFMHGPTFMANPLACAVALKSTQLLIEQDWQANIKRIEQQLSQYLMPLNQLDYVADVRVLGAIGVVELTFNVDMKTLQQQFVERGIWIRPFGKLVYVMPPYVITQQELSDLLEHLVEVVKTMQGAH

**>WP_003687694.1 *Neisseria gonorrhoeae***

MPSEHQHTSSLLNFDRTHLLHPYTSMTDPLPVYPVKRAEGVFIELADGTRLIDGMSSWWCAIHGYNHPVLNQAVENQMKQMAHVMFGGLTHEPAVELGKLLVGILPQGLDRIFYADSGSVSVEVALKMAVQYQQARGLTAKQNIATVRRGYHGDTWNAMSVCDPETGMHHIFGSALPQRYFVDNPKNRFDDEWDGADLQPVRALFEAHHVDIAAFILEPVVQGAGGMYFYHPQYLRGLRDLCDEFDIVLIFDEIATGFGRTGKMFACEHAEVVPDIMCIGKGLSGGYMTLAAAITSQKVTETISRGEAGVFMHGPTFMANPLACAVACASVKLLLSQDWQANIRRIESILKGRLKAAWDIRGVKDVRVLGAIGVIELEKGVDMARFQADCVAQGIWVRPFGRLVYLMPPYIISDGILTKLADKTVQILKEHSK

**>WP_015367618.1 *Enterobacter aerogenes***

MTLDDLAFDRRHIWHPYTSMTSPLPVYPVVSAHGCELSLAGGEQLIDGMSSWWAAIHGYNHPRLNAAMKAQIDQMSHVMFGGITHPSAVALCRQLVAMTPESLECVFLADSGSVAVEVAMKMALQYWQAKGQPRRRFLTFRNGYHGDTFGAMSVCDPQNSMHSLWQGYLPDNLFAPAPQSRFDGEWDEMDMVPFARLMAAHRHEIAAVILEPVVQGAGGMRMYHPEWLKRVRKMCDREGILLIADEIATGFGRTGKLFACEHAGISADILCLGKALTGGTMTLSAAITTRTVAETISNGEAGCFMHGPTFMGNPLACAVASESLRLLESGEWQQQVAAIEAQLKAELAPARESEWVADVRVLGAIGVVETRQPVNMAALQRFFVEQGVWIRPFGRLIYLMPPYIISPQQLTRLTRAVNMAVQEETFFSE

**>**[**WP_002895578.1**](https://www.ncbi.nlm.nih.gov/protein/488984790) ***Klebsiella pneumoniae***

MTLDDLAFDRRHIWHPYTSMTSPLPVYPVVSAHGCELSLAGGEQLVDGMSSWWAAIHGYNHPRLNAALKGQIDQMSHVMFGGITHPPAVALCRQLVAMTPASLECVFLADSGSVAVEVAMKMALQYWQAKGEPRRRFLTFRNGYHGDTFGAMSVCDPQNSMHSLWQGYLPDNLFAPAPQSRFDGEWDEMDMVPFARLMAAHRHEIAAVILEPIVQGAGGMRMYHPEWLKRVRKMCDREGILLIADEIATGFGRTGKLFACEHAGITADILCLGKALTGGTMTLSAAITTRTVAETISNGEAGCFMHGPTFMGNPLACAVAGESLRLLESGEWQPQVTAIEAQLQAELAPARGSALVADVRVLGAIGVVETRRPVNMAALQRFFVEQGVWIRPFGRLIYLMPPYIITPEQLTRLTRAVNQAVQDETFFSE

**>WP_003117900.1 *Pseudomonas aeruginosa***

MGLNADWMQRDLNVLWHPCTQMKDHERLPVIPIRRGEGVWLEDFEGKRYIDAVSSWWVNVFGHANPRINQRIKDQVDQLEHVILAGFSHQPVIELSERLVKITPPGLDRVFYADSGSAGIEVALKMSYHFWLNSGRPRKKRFVTLTNSYHGETIAAMSVGDVALFTETYKSLLLDTIKVPSPDCFLRPDGMCWEEHSRNMFAHMERTLAEGHDEIAAVIVEPLIQGAGGMRMYHPVYLKLLREACDRYGVHLIHDEIAVGFGRTGTMFACEQAGIAPDFLCLSKALTGGYLPMSAVLTSETVYRGFYDDYQTLRAFLHSHTYTGNPLACAAALATLDIFEEDKVIEANRALSTHMARATAHLADHPHVAEVRQTGMVLAIEMVQDKASRTPYPWQERRGLKVFQHGLERGALLRPLGSVVYFLPPYVITPEQIDFLAEVASEGIDIATRDAVSVAVSDFHPDHRDPG

**>WP_002370098.1 *Enterococcus faecalis***

MKYNYLVPMGDITKVNEHKTTIVRAEEEYVFDEEQKRFVDLRSGLWNTNLGYKKELYEIIRQRFTEQLSKSLTYLDIHSFHHPVYQEYAKKLATFADKEGFYEQVIYTNSGSECTELALKISRQINKSNQKILAFSQGYHGTFWGGMSISGLDQEVTDIYSPKLSNMEFIKSPENDIEEKNFFKHIEYHHHEYSAMIIEPVLGSAGIKMPSIRFLNKLGSLLKKYGIIVIFDEVATGFYRTGKPFYFHYLDFKPDIINLSKGINNGMLPFGVVLLSNDIVCELKKEKLEHFSTQNGNLLGVISAYETLNYYRQHEVEIVQNIQNLNELILTELNVYGISFRGIGCMFAIPIDDKQALPLIIQSLKQTGILCYQYFNSDEDNGLTLMPSFYTNYQKMLQIIRRIAKVVNAYA

**>WP_001110064.1 *Staphylococcus aureus***

MNYTQQLKQKDSEYVWHPFTQMGVYSKEEAIIIEKGKGSYLYDTNGNKYLDGYASLWVNVHGHNNKYLNKVIKKQLNKIAHSTLLGSSNIPSIELAEKLIEITPSNLRKVFYSDTGSASVEIAIKMAYQYWKNIDREKYAKKNKFITLNHGYHGDTIGAVSVGGIKTFHKIFKDLIFENIQVESPSFYRSNYDTENEMMTAILTNIEQILIERNDEIAGFILEPLIQGATGLFVHPKGFLKEVEKLCKKYDVLLICDEVAVGFGRTGKMFACNHEDVQPDIMCLGKAITGGYLPLAATLTSKKIYNAFLSDSHGVNTFFHGHTYTGNQIVCTVALENIRLYEKRKLLSHIETTSSTLEKQLHALKRHRNVGDVRGRGLMFGVELVTDKDSKTPLEIEKVERIVRNCKENGLMIRNLENVITFVPVLSMSNKEVKTMVRIFKKAVHNILDRKC

**Supplemental Figure S2A**. NCBI Reference Sequence for Mtb and ESKAPE (*Enterobacter aerogenes, Staphylococcus aureus, Klebsiella pneumoniae, Acinetobacter baumannii, Pseudomonas aeruginosa and Enterococcus faecalis*) BioA protein.

**Query: NP_216084.1 M. TUBERCULOSIS Query ID: lcl|Query_308311 Length: 437**

**Query range 1: 1 to 90**

**Query 11 EQIIAVDGAHLWHPYSSIGRE-AVSPVVAVA-A-HGAWLTLIRDGQPIEVLDAMSSWWTAIHGHG---HPALDQALTTQL-RVMNHVMFG 93**

**Query_308313 6 DLEHIWHPYTSMTQP-L--PTFKVKRA-YGATIEL-DDGRTL--IDGMSSWWCAIHGYN---HPELNQAVTDQL-QNMSHIMFG 78**

**Query_308316 6 LAFDRRHIWHPYTSMTSPLPVYPVVS---A-HGCELSLAGGEQ---LVDGMSSWWAAIHGYN---HPRLNAALKGQI-DQMSHVMFG 81**

**Query_308314 11 LLNFDRTHLLHPYTSMTDP-L--PVYPVKRA-EGVFIELA-DG--TRLIDGMSSWWCAIHGYN---HPVLNQAVENQM-KQMAHVMFG 87**

**Query_308315 6 LAFDRRHIWHPYTSMTSPLPVYPVVS---A-HGCELSLAGGEQ---LIDGMSSWWAAIHGYN---HPRLNAAMKAQI-DQMSHVMFG 81**

**Query_308317 15 LWHPCTQMKDH-ERLPVIPIRRG-EGVWL---EDFEGKRYIDAVSSWWVNVFGHA---NPRINQRIKDQV-DQLEHVILA 85**

**Query_308319 5 QQLKQKDSEYVWHPFTQMGVY-SKEEAIIIE-KGKGSYLY---DTNGNKYLDGYASLWVNVHGHN---NKYLNKVIKKQL-NKIAHSTLL 85**

**Query_308318 32 DEEQKRFVDLRSGLWNTNLGYKKELYEIIRQRFTEQLSKSLTYLDIH 78**

**Query range 2: 91 to 180**

**Query 94 GLTHEPAARLAKLLVDI--TPAGLDTVFFSDSGSVSVEVAAKMALQYWRG---RGLPGKRRLMTWRGGYHGDTFLAMSICDPHGGM---H 175**

**Query_308313 79 GLTHDPAIELGKILLKI--TPPSLDKIFYADSGSVAVEVALKMAVQFWTA---QGQPQKTNFITTRSGYHGDTWNAMSVCDPVTGM---H 160**

**Query_308316 82 GITHPPAVALCRQLVAM--TPASLECVFLADSGSVAVEVAMKMALQYWQA---KGEP-RRRFLTFRNGYHGDTFGAMSVCDPQNSM---H 162**

**Query_308314 88 GLTHEPAVELGKLLVGI--LPQGLDRIFYADSGSVSVEVALKMAVQYQQA---RGLTAKQNIATVRRGYHGDTWNAMSVCDPETGM---H 169**

**Query_308315 82 GITHPSAVALCRQLVAM--TPESLECVFLADSGSVAVEVAMKMALQYWQA---KGQP-RRRFLTFRNGYHGDTFGAMSVCDPQNSM---H 162**

**Query_308317 86 GFSHQPVIELSERLVKI--TPPGLDRVFYADSGSAGIEVALKMSYHFWLN---SGRPRKKRFVTLTNSYHGETIAAMSVGDV-------- 162**

**Query_308319 86 GSSNIPSIELAEKLIEI--TPSNLRKVFYSDTGSASVEIAIKMAYQYWKNIDREKYAKKNKFITLNHGYHGDTIGAVSV----GGIKTFH 169**

**Query_308318 79 SFHHPVYQEYAKKLATFADKEGFYEQVIYTNSGSECTELALKISRQINKS---N-----QKILAFSQGYHGTFWGGMSISGLDQEV---T 157**

**Query range 3: 181 to 270**

**Query 176 SLWTDVL--AAQVF--AP-QVP-------------R-DYDP------AYSAA----FEAQLAQHAGELAAVVVEPVVQGAGGMRFHDPRY 236**

**Query_308313 161 QIFGTSL--PNRLFVAAP-QTKFH-----------E-EWNQ------EDIAE----LEQAIQQNHENLAALIIEPIVQGAGGMRFYHPEY 225**

**Query_308316 163 SLWQGYL--PDNLF--AP-APQ-------------S-RFDG------EWDEMDMVPFARLMAAHRHEIAAVILEPIVQGAGGMRMYHPEW 227**

**Query_308314 170 HIFGSAL--PQRYF--VD-NPK-------------N-RFDD------EWDGADLQPVRALFEAHHVDIAAFILEPVVQGAGGMYFYHPQY 234**

**Query_308315 163 SLWQGYL--PDNLF--AP-APQ-------------S-RFDG------EWDEMDMVPFARLMAAHRHEIAAVILEPVVQGAGGMRMYHPEW 227**

**Query_308317 163 ALFTETY--KSLLL--DTIKVPSPDCFLRPDGMCWE-EHSR------NMFAH----MERTLAEGHDEIAAVIVEPLIQGAGGMRMYHPVY 237**

**Query_308319 170 KIFKDLIFENIQVE--SP-SFY-------------RSNYDTENEMMTAILTN----IEQILIERNDEIAGFILEPLIQGATGLFVHPKGF 239**

**Query_308318 158 DIYSPKL--SNMEF--IK-SPE-------------N-DIE---------EKN----FFKHIEYHHHEYSAMIIEPVL-GSAGIKMPSIRF 214**

**Query range 4: 271 to 360**

**Query 237 LHDLRDICRRYEVLLIFDEIATGFGRTGALFAADHAGVSPDIMCVGKALTGGYLSLAATLCTADVAHTI--SAGAAG--ALMHGPTFMAN 322**

**Query_308313 226 LRQAKALCEKYHLLLIFDEIATGFGRTGKLFAWEHAQVEPDIMCLGKGLTGGYMTLSATLTTKHVAETI--SRGEAG--VFMHGPTFMAN 311**

**Query_308316 228 LKRVRKMCDREGILLIADEIATGFGRTGKLFACEHAGITADILCLGKALTGGTMTLSAAITTRTVAETI--SNGEAG--CFMHGPTFMGN 313**

**Query_308314 235 LRGLRDLCDEFDIVLIFDEIATGFGRTGKMFACEHAEVVPDIMCIGKGLSGGYMTLAAAITSQKVTETI--SRGEAG--VFMHGPTFMAN 320**

**Query_308315 228 LKRVRKMCDREGILLIADEIATGFGRTGKLFACEHAGISADILCLGKALTGGTMTLSAAITTRTVAETI--SNGEAG--CFMHGPTFMGN 313**

**Query_308317 238 LKLLREACDRYGVHLIHDEIAVGFGRTGTMFACEQAGIAPDFLCLSKALTGGYLPMSAVLTSETVYRGFYDDYQTLR--AFLHSHTYTGN 325**

**Query_308319 240 LKEVEKLCKKYDVLLICDEVAVGFGRTGKMFACNHEDVQPDIMCLGKAITGGYLPLAATLTSKKIYNAF--LSDSHGVNTFFHGHTYTGN 327**

**Query_308318 215 LNKLGSLLKKYGIIVIFDEVATGFYRTGKPFYFHYLDFKPDIINLSKGINNGMLPFGVVLLSNDIV--------------------------280**

**Query range 5: 361 to 450**

**Query 323 PLACAVSVASVELLLGQDWRTRITELAAGLTAGLDTARA----LPAVTDVRVCGAIGVIECDRPVDLAVATPA-------------ALDR 395**

**Query_308313 312 PLACAVALKSTQLLIEQDWQANIKRIEQQLSQYLMPLNQ----LDYVADVRVLGAIGVVELTFNVDMKTLQQQ-------------FVER 384**

**Query_308316 314 PLACAVAGESLRLLESGEWQPQVTAIEAQLQAELAPARG----SALVADVRVLGAIGVVETRRPVNMAALQRF-------------FVEQ 386**

**Query_308314 321 PLACAVACASVKLLLSQDWQANIRRIESILKGRLKAAWD----IRGVKDVRVLGAIGVIELEKGVDMARFQAD-------------CVAQ 393**

**Query_308315 314 PLACAVASESLRLLESGEWQQQVAAIEAQLKAELAPARE----SEWVADVRVLGAIGVVETRQPVNMAALQRF-------------FVEQ 386**

**Query_308317 326 PLACAAALATLDIFE----EDKVIEANRALSTHMARATAHLADHPHVAEVRQTGMVLAIEMVQ--DKASRTPYPWQERRGLKVFQHGLER 409**

**Query_308319 328 QIVCTVALENIRLYEKRKLLSHIETTSSTLEKQLHALKR----HRNVGDVRGRGLMFGVEL--VTDKDSKTPL-------------EIEK 398**

**Query_308318 ------------------------------------------------------------------------------------------**

**Query range 6: 451 to 500**

**Query 396 -----------GVWLRPFRNLVYAMPPYICTPAEI---TQITSAMVEVAR 431**

**Query_308313 385 -----------GIWIRPFGKLVYVMPPYVITQQEL---SDLLEHLVEVVK 420**

**Query_308316 387 -----------GVWIRPFGRLIYLMPPYIITPEQL---TRLTRAVNQAVQ 422**

**Query_308314 394 -----------GIWVRPFGRLVYLMPPYIISDGIL---TKLADKTVQILK 429**

**Query_308315 387 -----------GVWIRPFGRLIYLMPPYIISPQQL---TRLTRAV----- 417**

**Query_308317 410 -----------GALLRPLGSVVYFLPPYVITPEQIDFLAEVASEGIDIA- 447**

**Query_308319 399 VERIVRNCKENGLMIRNLENVITFVPVLSMSNKEV--------------- 433**

**Query_308318 -----------------------------------------------------**

**Supplemental Figure S2B**. Sequence alignment between *Mt*BioA and ESKAPE BioA

**Supplemental Table S5**. Sequence alignment between *Mt*BioA and ESKAPE BioA

# blastp

# Iteration: 0

# Query: **NP_216084.1 *M. tuberculosis***

# RID: PEJY8RH2114

# Database: n/a

# Fields:

| Query | Subject acc.vear. | % Identity | length | Mismatches | Gap opens | q. start | q. end | s. start | s. end | Evalue | Bit score | Positives |
| --- | --- | --- | --- | --- | --- | --- | --- | --- | --- | --- | --- | --- |
| *A. baumannii* | WP_000131427.1 | 51.429 | 420 | 194 | 6 | 17 | 431 | 6 | 420 | 4.02e-157 | 441 | 68.10 |
| *K. pneumoniae* | WP_002895578.1 | 52.009 | 423 | 192 | 5 | 14 | 431 | 6 | 422 | 1.09e-152 | 430 | 68.09 |
| *N. gonorrhoeae* | WP_003687694.1 | 50.708 | 424 | 199 | 5 | 13 | 431 | 11 | 429 | 2.20e-150 | 424 | 66.27 |
| *E. aerogenes* | WP_015367618.1 | 51.675 | 418 | 191 | 5 | 14 | 426 | 6 | 417 | 2.32e-150 | 424 | 67.70 |
| *P. aeruginosa* | WP_003117900.1 | 36.242 | 447 | 234 | 11 | 21 | 430 | 15 | 447 | 1.45e-85 | 259 | 53.69 |
| *S. aureus* | WP_001110064.1 | 31.963 | 438 | 260 | 11 | 11 | 419 | 5 | 433 | 2.50e-81 | 248 | 52.28 |
| *E.* *faecalis* | WP_002370098.1 | 24.806 | 258 | 179 | 6 | 51 | 302 | 32 | 280 | 4.64e-25 | 95.5 | 45.35 |

**Supplemental Table S6**. Analysis of active site between *Mt*BioA and ESKAPE BioA

| **Query number** | **Organism/accession** | **Identity of active site residues** | **Number of mutations and deletions among the 31 residues with 5Å of ligand** |
| --- | --- | --- | --- |
| **Query** | *M. tuberculosis/*  WP_003687694.1 | n.a. | n.a. |
| **Query_308313** | *A. baumannii/*  WP_015367618.1 | G155S, G172T, R403G | 3 nonsynonymous mutations (all and non-conservative) |
| **Query_308316** | *Klebsiella pneumoniae/*  WP_002895578.1 | G155N, G172N, G173S, R403G | 4 nonsynonymous mutations (all and non-conservative) |
| **Query_308314** | *Neisseria gonorrhoeae/*  WP_003687694.1 | G155R, G172T, R403G | 3 nonsynonymous mutations (all and non-conservative) |
| **Query_308315** | *Enterobacter aerogenes/* WP_015367618.1 | G155N, G172N, G173S, R403G | 4 nonsynonymous mutations |
| **Query_308317** | *Pseudomonas aeruginosa/* WP_003117900.1 | Y25C, M91I, F92L, G93A, G155N, G156S, C168G, P170V, G172-, G173-, M174-, M175-, M314L, G316S, P317H, M320T, F402L, R403G | 14 nonsynonymous mutations (3 conservative and 11 non-conservative),  4 deletions |
| **Query_308319** | *Staphylococcus aureus/*  WP_001110064.1 | Y21F, W64L, M91T, F92L, G93L, G155H, M165V, C168-, D169-, P170-, M174I, G227T, I256V, M314F, P317H, M320T, F402L, R403E, | 15 nonsynonymous mutations (3 conservative and 12 non-conservative),  3 deletions |
| **Query_308318** | *Enterococcus faecalis/*  WP_002370098.1 | P24-, Y25-, W64L, M91D, F92I, G93H, G155Q, C168S, D169G, P170L, G172Q, G173E, M174V, H175T, A226S, G227A, I256V, M314-, G316-, P317-, T318-, M320-, R400-, F402-, R403-, Y407- | 15 nonsynonymous mutations (3 conservative and 12 non-conservative),  11 deletions |

Amino acid residues within 5 Å of *M. tuberculosis* BioA active site

- Red is the bromo-fluorophenyl
- orange is the piperazine
- green is the azaindolone
- blue interacts with PLP cofactor

P24, **Y25**, **W64, W65**, M91, F92, G93; G155, G156, **Y157,** M165, C168, D169, P170, G172, G173, M174, H175, A226, G227, **I256**, **K283,** M314, G316, P317, T318, M320, R400, **F402**, R403, **Y407**

|  |
| --- |

# **Supplemental Table S7.** **C48** Resistant Mutants and AA change

| Sample | Class | AA change | MIC shift |
| --- | --- | --- | --- |
| 1 | High-level resistance 1 (FOR10) | Met91Ile | Large |
| 2 | High-level resistance 2 (FOR22) | Met91Ile | Large |
| 3 | High-level resistance 4 (KK1) | Met91Ile | Large |
| 4 | Medium-level resistance 4 (KK2) | Met91Val | Medium |
| 5 | High-level resistance 5 (KK3) | Met91Ile | Large |
| 6 | High-level resistance 6 (KK4) | Met91Ile | Large |
| 7 | Medium-level resistance 1 (FA5) | Met91Val | Medium |
| 8 | High-level resistance 7 (FA9) | Met91Ile | Large |
| 9 | Medium-level resistance 5 (FA13) | Met91Thr | Medium |
| 10 | High-level resistance 3 (FA19) | Met91Ile | Large |
| 11 | High-level resistance 8 (FA23) | Met91Ile | Large |
| 12 | Medium-level resistance 6 (FA30) | Met91Thr | Medium |
| 13 | Medium-level resistance 2 (FA35) | Met91Val | Medium |
| 14 | High-level resistance 9 (FA37) | Met91Ile | Large |
| 15 | High-level resistance 10 (FA44) | Met91Ile | Large |
| 16 | Medium-level resistance 3 (FA49) | Cys168Tyr | Medium |
| FOR = resistance mutants were generated from frequency of resistance assay. FA = resistance mutants were generated from fluctuation assay. KK = resistance mutants were generated from kill kinetics study. AA change = amino acid change. MIC shift = MIC shift compared to WT. | | | |

# **Supplemental Figure S3.** Spleen efficacy of **C48** in conventional mice model


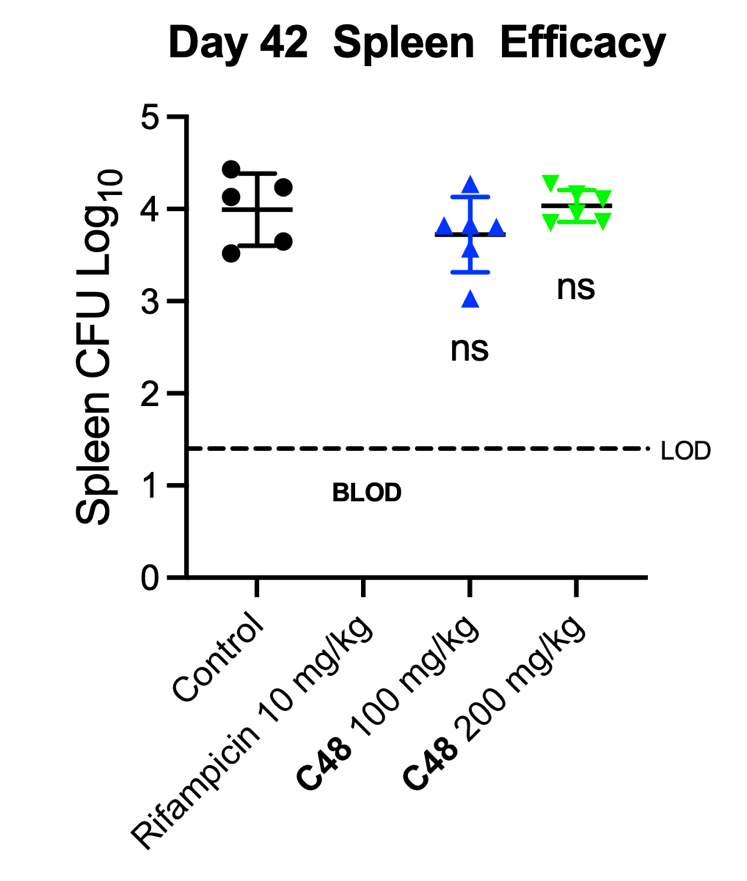


**Supplemental Figure S3. C48** was evaluated in a standard acute model of TB infection in BALB/c. The mice (each group contains 5 mice) were infected at day 0, drugs were administrated 14 days post infection via oral gavage 5 days/week, and the treatment lasted for 28 days. Spleen data was collected and CFU was counted at Day 42, LOD = limit of detection, BLOD = below the limit of detection.

# **Chemistry**

## **^1^H and ^13^C NMR Spectra**

## **HSQC of C48**


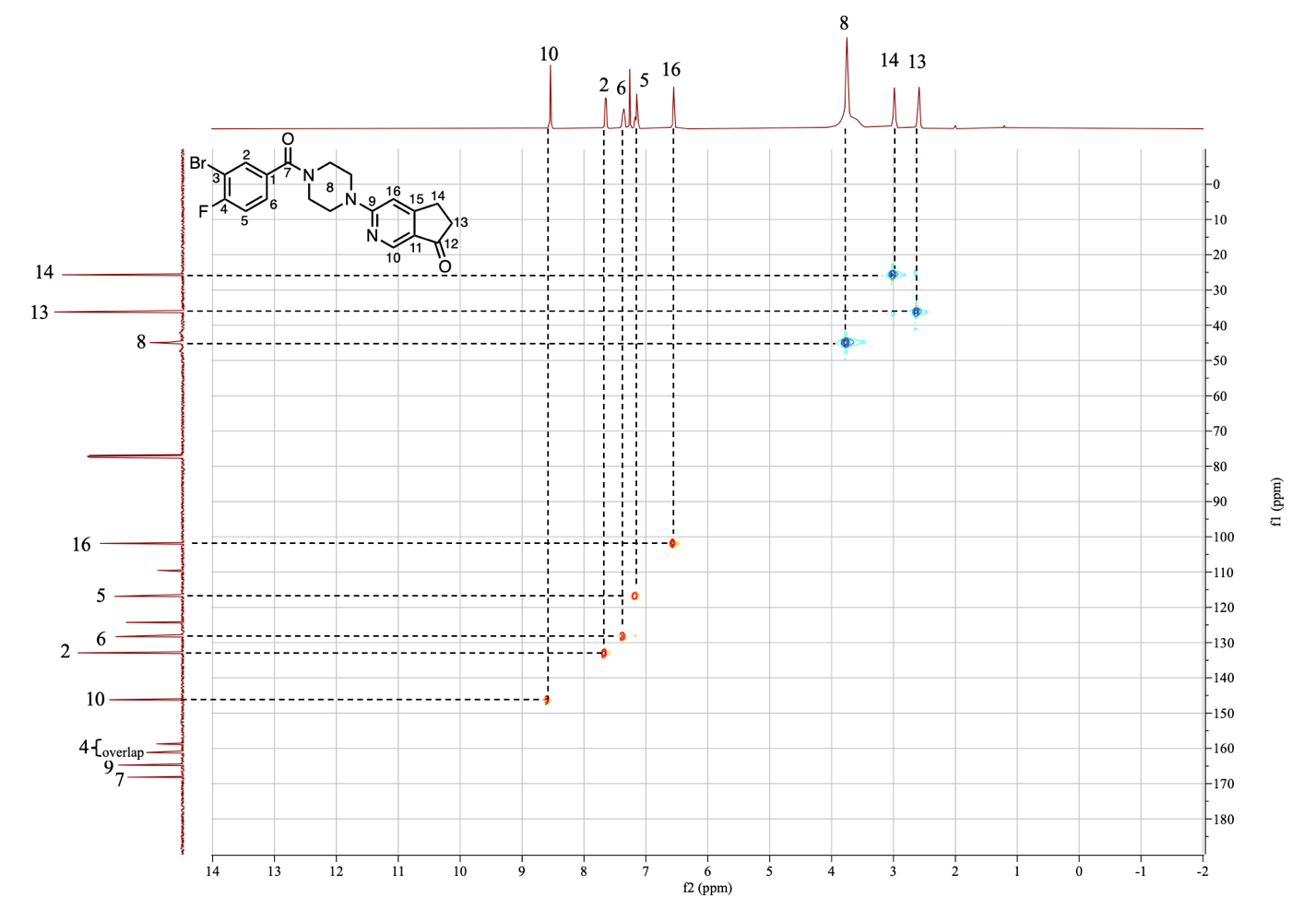


## **^19^F NMR of C48**

## **HPLC Trace of C21**


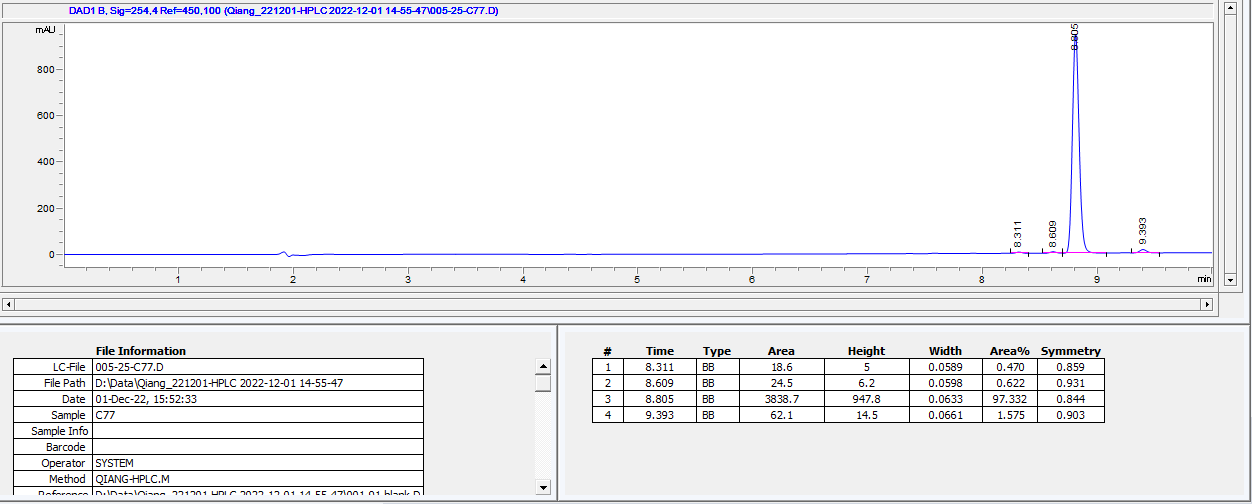


## **HPLC Trace of C48**


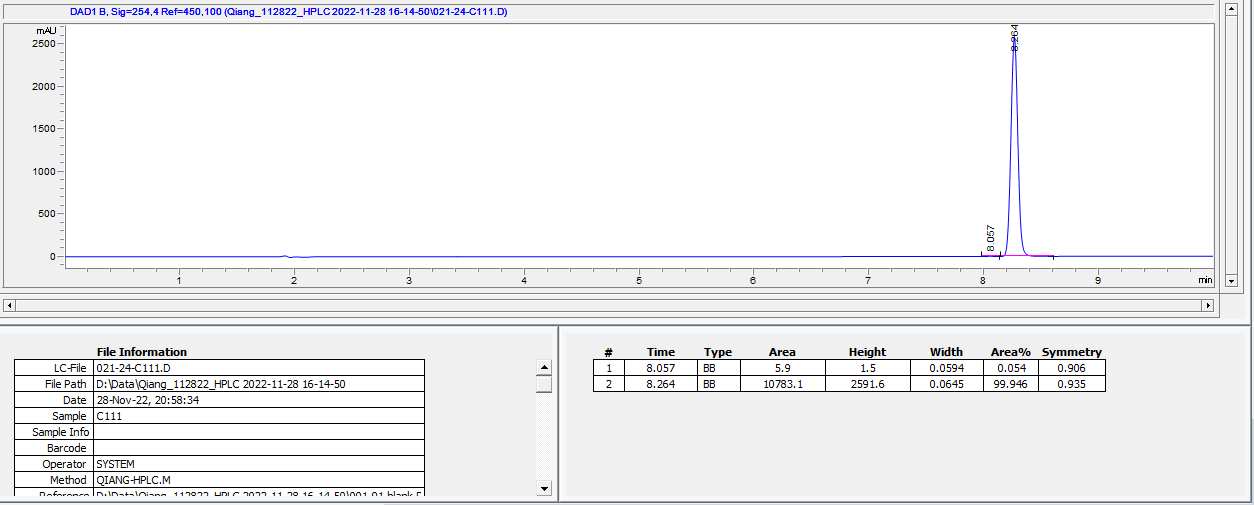


## **HRMS of C21**


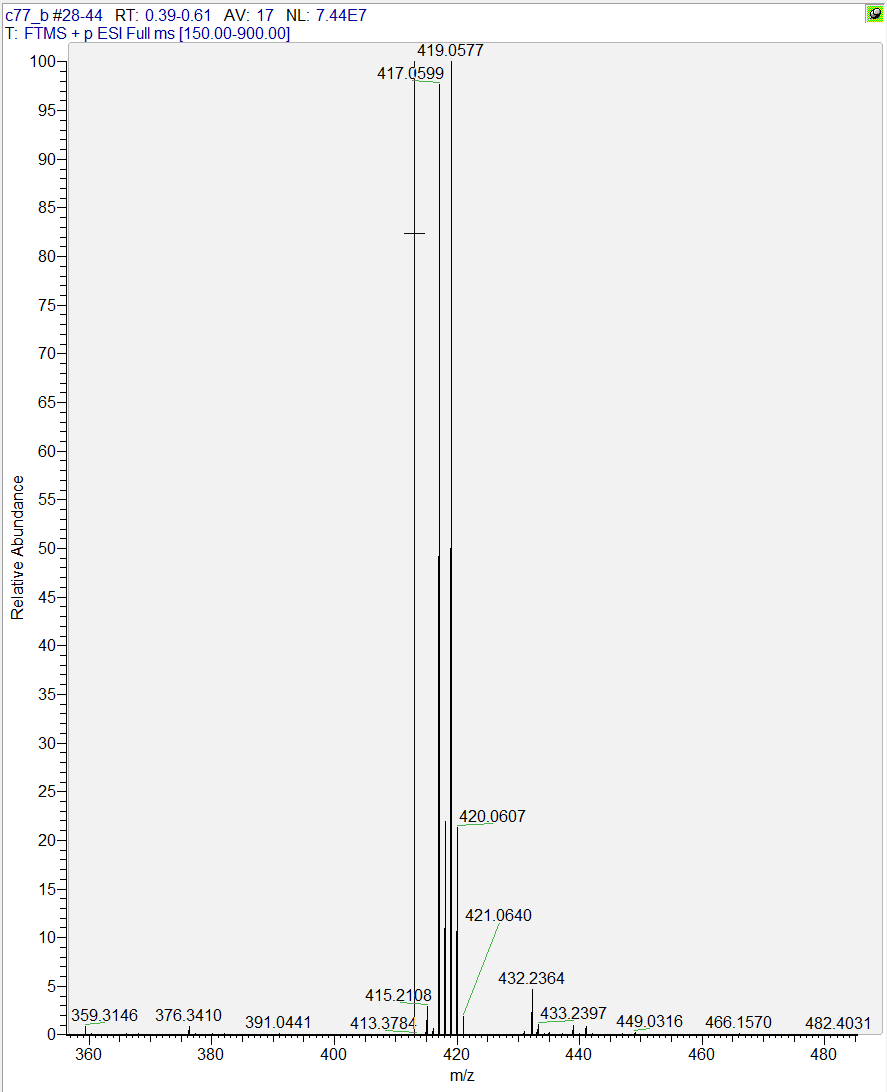


## **HRMS of compound 10**


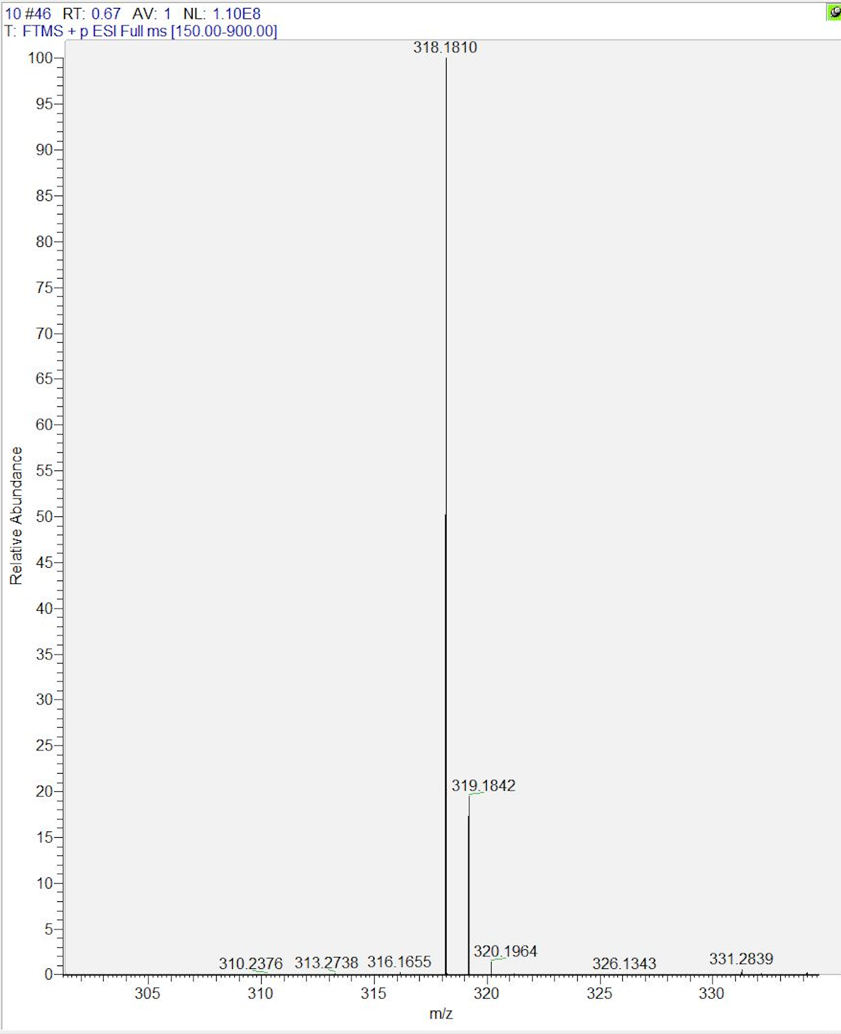


## **HRMS of C48**


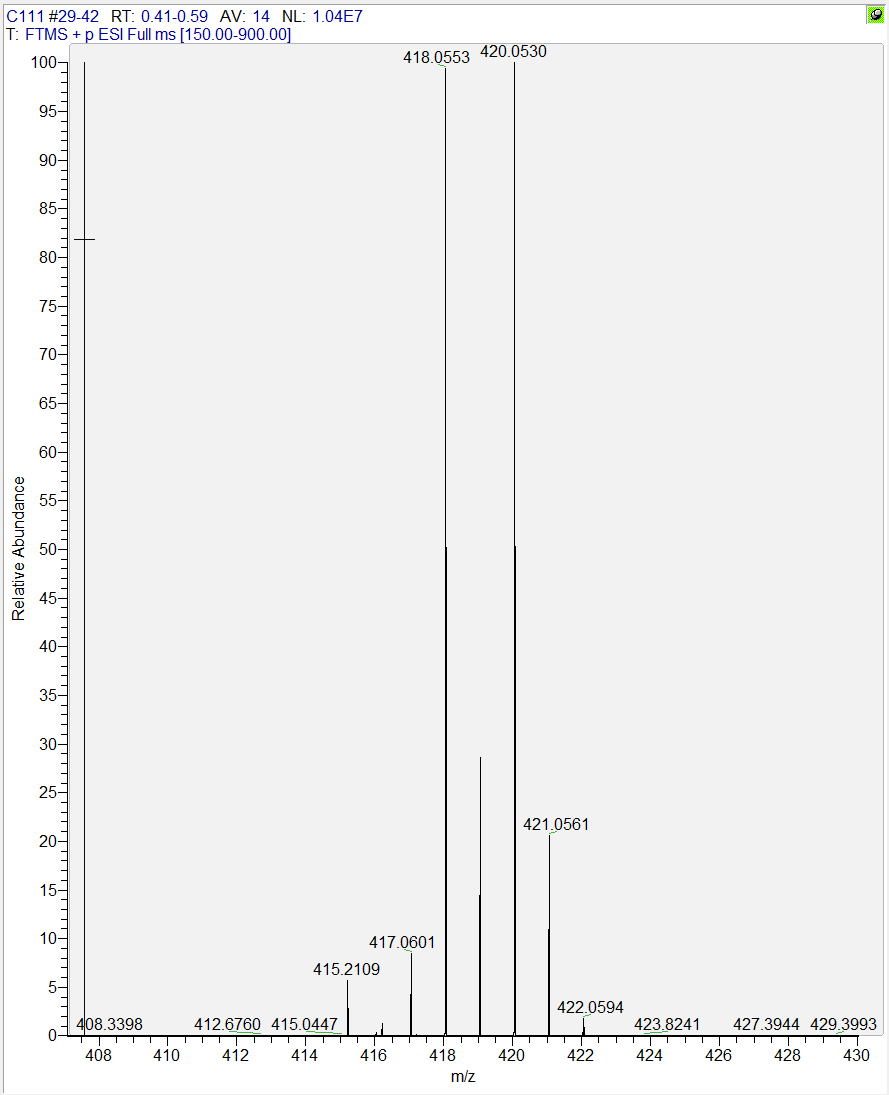


(1) Liu, F.; Dawadi, S.; Maize, K. M.; Dai, R.; Park, S. W.; Schnappinger, D.; Finzel, B. C.; Aldrich, C. C., Structure-Based Optimization of Pyridoxal 5 '-Phosphate-Dependent Transaminase Enzyme (BioA) Inhibitors that Target Biotin Biosynthesis in Mycobacterium tuberculosis. *J. Med. Chem.* **2017**, *60* (13), 5507-5520.

(2) Carfrae, L. A.; MacNair, C. R.; Brown, C. M.; Tsai, C. N.; Weber, B. S.; Zlitni, S.; Rao, V. N.; Chun, J.; Junop, M. S.; Coombes, B. K.; Brown, E. D., Mimicking the human environment in mice reveals that inhibiting biotin biosynthesis is effective against antibiotic-resistant pathogens. *Nat. Microbiol.* **2020**, *5* (1), 93-101.

(3) Dai, R.; Wilson, D. J.; Geders, T. W.; Aldrich, C. C.; Finzel, B. C., Inhibition of Mycobacterium tuberculosis transaminase BioA by aryl hydrazines and hydrazides. *Chembiochem* **2014**, *15* (4), 575-586.

(4) Zlitni, S.; Ferruccio, L. F.; Brown, E. D., Metabolic suppression identifies new antibacterial inhibitors under nutrient limitation. *Nat. Chem. Biol.* **2013**, *9* (12), 796-804.

(5) Mann, S.; Ploux, O., 7,8-Diaminoperlargonic acid aminotransferase from Mycobacterium tuberculosis, a potential therapeutic target. Characterization and inhibition studies. *FEBS J.* **2006**, *273* (20), 4778-4789.

(6) Bockman, M. R.; Engelhart, C. A.; Cramer, J. D.; Howe, M. D.; Mishra, N. K.; Zimmerman, M.; Larson, P.; Alvarez-Cabrera, N.; Park, S. W.; Boshoff, H. I. M.; Bean, J. M.; Young, V. G., Jr.; Ferguson, D. M.; Dartois, V.; Jarrett, J. T.; Schnappinger, D.; Aldrich, C. C., Investigation of ( S)-(-)-Acidomycin: A Selective Antimycobacterial Natural Product That Inhibits Biotin Synthase. *ACS Infect. Dis.* **2019**, *5* (4), 598-617.

(7) Eisenberg, M. A.; Hsiung, S. C., Mode of action of the biotin antimetabolites actithiazic acid and alpha-methyldethiobiotin. *Antimicrob. Agents Chemother.* **1982**, *21* (1), 5-10.

(8) Lin, S.; Cronan, J. E., The BioC O-methyltransferase catalyzes methyl esterification of malonyl-acyl carrier protein, an essential step in biotin synthesis. *J. Biol. Chem.* **2012**, *287* (44), 37010-37020.

(9) Alexeev, D.; Baxter, R. L.; Campopiano, D. J.; McAlpine, R. S.; McIver, L.; Sawyer, L., Rational design of an inhibitor of dethiobiotin synthetase; Interaction of 6-hydroxypyrimidin-4(3H)-one with the adenine base binding site. *Tetrahedron* **1998**, *54* (52), 15891-15898.

(10) Ploux, O.; Breyne, O.; Carillon, S.; Marquet, A., Slow-binding and competitive inhibition of 8-amino-7-oxopelargonate synthase, a pyridoxal-5'-phosphate-dependent enzyme involved in biotin biosynthesis, by substrate and intermediate analogs. Kinetic and binding studies. *Eur J Biochem* **1999**, *259* (1-2), 63-70.

(11) Dunn, P. L.; North, R. J., Virulence ranking of some Mycobacterium tuberculosis and Mycobacterium bovis strains according to their ability to multiply in the lungs, induce lung pathology, and cause mortality in mice. *Infect. Immun.* **1995**, *63* (9), 3428-3437.

(12) Woong Park, S.; Klotzsche, M.; Wilson, D. J.; Boshoff, H. I.; Eoh, H.; Manjunatha, U.; Blumenthal, A.; Rhee, K.; Barry, C. E., 3rd; Aldrich, C. C.; Ehrt, S.; Schnappinger, D., Evaluating the sensitivity of Mycobacterium tuberculosis to biotin deprivation using regulated gene expression. *Plos Pathog.* **2011**, *7* (9), e1002264.

(13) Malherbe, S. T.; Chen, R. Y.; Yu, X.; Smith, B.; Liu, X.; Gao, J.; Diacon, A. H.; Dawson, R.; Tameris, M.; Zhu, H.; Qu, Y.; Jin, H.; Pan, S.; Dodd, L. E.; Wang, J.; Goldfeder, L. C.; Cai, Y.; Arora, K.; Vincent, J.; Narunsky, K.; Serole, K.; Goliath, R. T.; Da Costa, L.; Taliep, A.; Aziz, S.; Daroowala, R.; Thienemann, F.; Mukasa, S.; Court, R.; Sossen, B.; Ahlers, P.; Mendelsohn, S. C.; White, L.; Gouel, A.; Lau, C. Y.; Hassan, S.; Liang, L.; Duan, H.; Moghaddam, G. K.; Paripati, P.; Lahouar, S.; Harris, M.; Wollenberg, K.; Jeffrey, B.; Tartakovsky, M.; Rosenthal, A.; Duvenhage, M.; Armstrong, D. T.; Song, T.; Winter, J.; Gao, Q.; Via, L. E.; Wilkinson, R. J.; Walzl, G.; Barry, C. E., 3rd, PET/CT guided tuberculosis treatment shortening: a randomized trial. *medRxiv* **2024**.

(14) Song, T.; Park, Y.; Shamputa, I. C.; Seo, S.; Lee, S. Y.; Jeon, H. S.; Choi, H.; Lee, M.; Glynne, R. J.; Barnes, S. W.; Walker, J. R.; Batalov, S.; Yusim, K.; Feng, S.; Tung, C. S.; Theiler, J.; Via, L. E.; Boshoff, H. I.; Murakami, K. S.; Korber, B.; Barry, C. E., 3rd; Cho, S. N., Fitness costs of rifampicin resistance in Mycobacterium tuberculosis are amplified under conditions of nutrient starvation and compensated by mutation in the beta' subunit of RNA polymerase. *Mol. Microbiol.* **2014**, *91* (6), 1106-19.

(15) Bang, H.; Park, S.; Hwang, J.; Jin, H.; Cho, E.; Kim, D. Y.; Song, T.; Shamputa, I. C.; Via, L. E.; Barry, C. E.; Cho, S. N.; Lee, H., Improved rapid molecular diagnosis of multidrug-resistant tuberculosis using a new reverse hybridization assay, REBA MTB-MDR. *J. Med. Microbiol.* **2011**, *60* (Pt 10), 1447-1454.

(16) Akusobi, C.; Choudhery, S.; Benghomari, B. S.; Wolf, I. D.; Singhvi, S.; Ioerger, T. R.; Rubin, E. J., Transposon-sequencing across multiple Mycobacterium abscessus isolates reveals significant functional genomic diversity among strains. *mBio* **2025**, *16* (2), e0337624.

(17) Liu, Q.; Engelhart, C. A.; Wallach, J. B.; Tiwari, D.; Ge, P.; Manna, A.; Panda, S.; McCue, W. M.; Wong, T. Y.; Sharma, S.; Jayasinghe, Y. P.; Fuller, J.; Ronning, D. R.; Bockman, M. R.; Cheung, A.; Dartois, V.; Zimmerman, M. D.; Schnappinger, D.; Aldrich, C. C., Metabolically Stable Adenylation Inhibitors of Biotin Protein Ligase as Antibacterial Agents. *J. Med. Chem.* **2025**, *68* (3), 3065-3087.
