# Supplementary material for: Structure-Guided Development of a Potent BioA Inhibitor Validates Biotin Synthesis Inhibition as a Therapeutic Strategy for Tuberculosis
Source: bioRxiv. 2025 Sep 24:2025.09.24.678246. Preprint. [Version 1] doi: 10.1101/2025.09.24.678246 (PMC12485673; doi:10.1101/2025.09.24.678246)
Supplement: Supplement 2 [file media-2.docx]

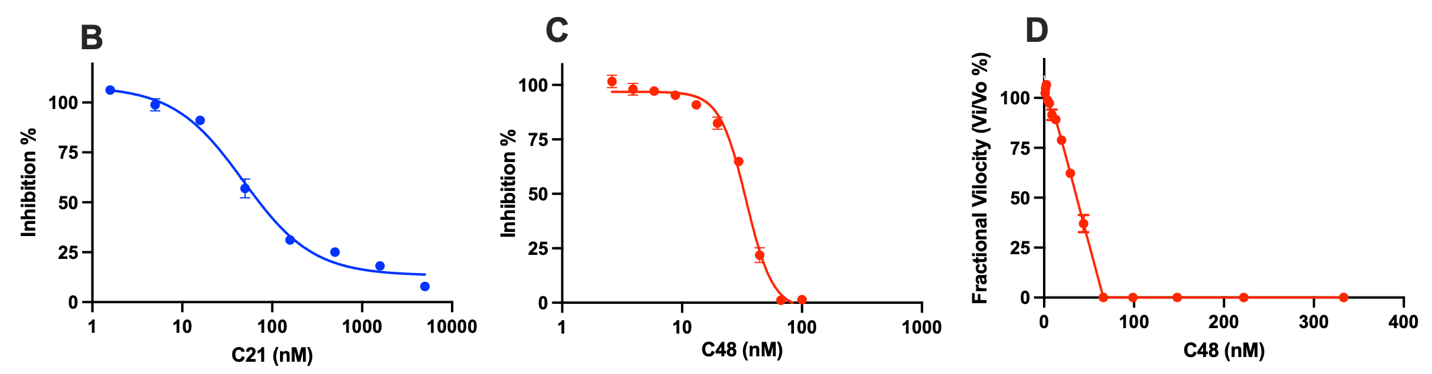


**Extended Data Fig. 1** **Biochemical Characterization of BioA inhibitors** (**A**) Biotin biosynthesis in *Mtb*. Pimeloyl-ACP (**1**) is converted to 7- keto-8-aminopelargonic acid (KAPA, **2**) by BioF (KAPA synthetase). Transamination by BioA (DAPA synthetase) converts 2 to 7,8-diaminopelargonic acid (DAPA, **3**), followed by insertion of a carbonyl by BioD (dethiobiotin synthetase) gives rise to dethiobiotin (DTB, **4**). Finally, BioB (biotin synthase) is responsible for the conversion of 4 to biotin (5). (**B**) and (**C**) The biochemical activity of **C21** and **C48**. The assay was performed with **C21** (3-fold dilution ranging from 5000 to 1.6 nM) or **C48** (1.5-fold dilution ranging from 100 to 2.6 nM) in 50 nM BioA, 320 nM BioD, 3 µM KAPA, 1 mM S-adenosyl methionine, 20 nM Fluorescent-DTB tracer, 35 nM streptavidin, 5 mM ATP, 50 mM NaHCO_3_, 1 mM MgCl_2_, 0.1 mM PLP, 0.0025% Igepal CA630, and 100 mM Bicine [pH 8.6]. (**D**) Linear titration of activity with **C48**. Initial *K*i was explored with **C48** (1.5-fold dilution ranging from 333 to 1.1 nM) in 50 nM BioA, 320 nM BioD, 3 µM KAPA, 1 mM S-adenosyl methionine, 20 nM Fluorescent-DTB tracer, 35 nM streptavidin, 5 mM ATP, 50 mM NaHCO_3_, 1 mM MgCl_2_, 0.1 mM PLP, 0.0025% Igepal CA630, and 100 mM Bicine [pH 8.6].


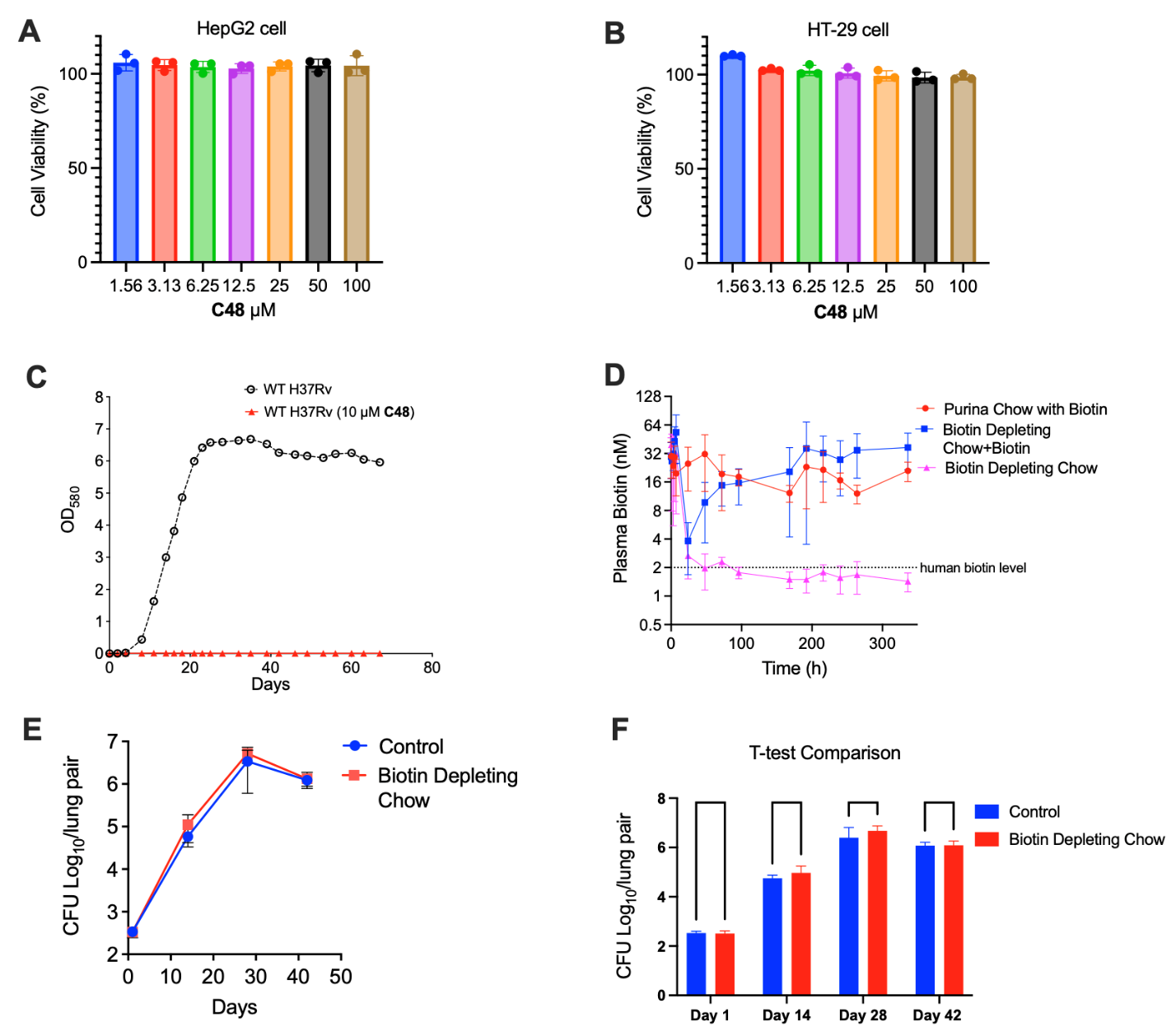


﻿**Extended Data Fig. 2 ﻿Biotin depletion did not impair the growth of *Mtb* in vivo**  (**A**) and (**B**) Viability of HepG2 and HT-29 after 48 h treatment with the indicated concentrations of **C48**. Values are normalized to vehicle treated condition (DMSO). The assay was performed as triplicate. (**C**) OD readings of **C48** kill kinetics, the assay was performed in GAST medium against wild-type (WT) *Mtb* (H37Rv) and the OD_580_ was taken at the indicated time. (**D**) ﻿Quantification of biotin levels in mice plasma following different mice chow administrated, Purina Chow : regular mice chow with biotin supplemented (PicoLab® Rodent Diet 20), Biotin depletion chow (biotin-free, Inotiv TD.81079). 6 mice were tracked for biotin levels for 2 weeks and due to IACUC guidelines, timepoints at 1, 3, 5, 7, 48, 72 and 96 h contained 3 mice samples and the rest timepoints contained 6 mice samples. (**E**) and (**F**) Biotin depletion ﻿has no effect on the ability of WT *Mtb* to cause infection in vivo. Mice (n=5) were infected with WT *Mtb* under regular chow and biotin depletion chow. The lungs were collected, homogenized and the CFU was counted at the indicated time.

**Extended Data** **Table 1. Pharmacokinetics of 6, C21 and C48 in CD-1 mice.**

| Compd | i.v. PK parameters^a^ | | | | p.o. PK parameters^b^ | | |
| --- | --- | --- | --- | --- | --- | --- | --- |
|  | AUC_0–∞_ (*μ*g·hr/mL) | *V*d (L/kg) | CL (mL/kg·min) | *t*_1/2_ (h) | AUC_0–∞_ (*μ*g·hr/mL) | *F* (%) | AUC (p.o.)/MIC |
| **6** | 0.3 | 0.54 | 30.6 | 0.2 | 0.46 | 3.4 | 0.04 |
| **C21** | 4.0 | 0.37 | 5.3 | 0.82 | 108 | 100 | 57 |
| **C48^c^** | 3.3 | 0.26 | 3.2 | 0.91 | 146 | 100 | 1570 |

^a^i.v. dose (D_iv_) = 5 mg/kg, ^b^p.o. dose (D_po_) = 25 mg/kg. AUC_0–∞_, area under the plasma concentration−time curve from time 0 to infinity; *V*d, volume of distribution; CL, clearance; *t*_1/2_, terminal elimination half-life; *F*, relative oral bioavailability calculated as follows: *F* = 100 × [(AUC_po_ × D_iv_)/(AUC_iv_ × D_po_); AUC (p.o.)/MIC = AUC_0–∞_(p.o.)/MIC_50_]. ^c^The i.v. dose of **C48** was reduced to 0.648 mg/kg due to solubility issue. Each group contains 3 mice.
