# Supplementary material for: Structure-Guided Development of a Potent BioA Inhibitor Validates Biotin Synthesis Inhibition as a Therapeutic Strategy for Tuberculosis
Source: bioRxiv. 2025 Sep 24:2025.09.24.678246. Preprint. [Version 1] doi: 10.1101/2025.09.24.678246 (PMC12485673; doi:10.1101/2025.09.24.678246)
Supplement: Supplement 4 [file media-4.pdf]

Full wwPDB X-ray Structure Validation Report (i)

Jul 30, 2025 – 02:45 PM EDT

PDB ID	:	9D7M / pdb_00009d7m
Title	:	Crystal structure of Mycobacterium tuberculosis 7,8-diaminopelargonic acid synthase (BioA) in complex with inhibitor C48
Deposited on	:	2024-08-16
Resolution	:	1.97 Å(reported)

This wwPDB validation report is for manuscript review

This is a Full wwPDB X-ray Structure Validation Report.

This report is produced by the wwPDB biocuration pipeline after annotation of the structure.

We welcome your comments at *validation@mail.wwpdb.org*
A user guide is available at
https://www.wwpdb.org/validation/2017/XrayValidationReportHelp
with specific help available everywhere you see the (i) symbol.

The types of validation reports are described at
http://www.wwpdb.org/validation/2017/FAQs#types.

For Manuscript Review

The following versions of software and data (see references (i)) were used in the production of this report:

MolProbity	:	4-5-2 with Phenix2.0rc1
Mogul	:	2022.3.0, CSD as543be (2022)
Xtriage (Phenix)	:	2.0rc1
EDS	:	3.0
buster-report	:	1.1.7 (2018)
Percentile statistics	:	20231227.v01 (using entries in the PDB archive December 27th 2023)
CCP4	:	9.0.006 (Gargrove)
Density-Fitness	:	1.0.12
Ideal geometry (proteins)	:	Engh & Huber (2001)

1 Overall quality at a glance (i)

The following experimental techniques were used to determine the structure:
X-RAY DIFFRACTION

The reported resolution of this entry is 1.97 Å.

Percentile scores (ranging between 0-100) for global validation metrics of the entry are shown in the following graphic. The table shows the number of entries on which the scores are based.

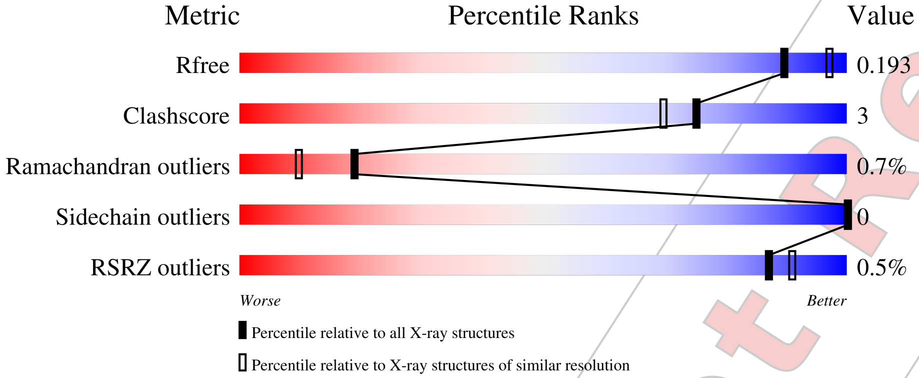

Metric	Whole archive (#Entries)	Similar resolution (#Entries, resolution range(Å))
R_{free}	164625	1356 (1.98-1.98)
Clashscore	180529	1437 (1.98-1.98)
Ramachandran outliers	177936	1426 (1.98-1.98)
Sidechain outliers	177891	1426 (1.98-1.98)
RSRZ outliers	164620	1356 (1.98-1.98)

The table below summarises the geometric issues observed across the polymeric chains and their fit to the electron density. The red, orange, yellow and green segments of the lower bar indicate the fraction of residues that contain outliers for >=3, 2, 1 and 0 types of geometric quality criteria respectively. A grey segment represents the fraction of residues that are not modelled. The numeric value for each fraction is indicated below the corresponding segment, with a dot representing fractions <=5% The upper red bar (where present) indicates the fraction of residues that have poor fit to the electron density. The numeric value is given above the bar.

Mol	Chain	Length	Quality of chain
1	A	429	91% 9%
1	B	429	92% 8%

Ideal geometry (DNA, RNA) : Parkinson et al. (1996)
Validation Pipeline (wwPDB-VP) : 2.45.1

For Manuscript Review

2 Entry composition (i)

There are 4 unique types of molecules in this entry. The entry contains 7480 atoms, of which 0 are hydrogens and 0 are deuteriums.

In the tables below, the ZeroOcc column contains the number of atoms modelled with zero occupancy, the AltConf column contains the number of residues with at least one atom in alternate conformation and the Trace column contains the number of residues modelled with at most 2 atoms.

- Molecule 1 is a protein called Adenosylmethionine-8-amino-7-oxononanoate aminotransferase.

Mol	Chain	Residues	Atoms					ZeroOcc	AltConf	Trace
1	A	429	Total 3228	C 2055	N 569	O 583	S 21	0	5	0
1	B	429	Total 3244	C 2062	N 574	O 586	S 22	0	6	0

- Molecule 2 is PYRIDOXAL-5'-PHOSPHATE (CCD ID: PLP) (formula: $C_8H_{10}NO_6P$).

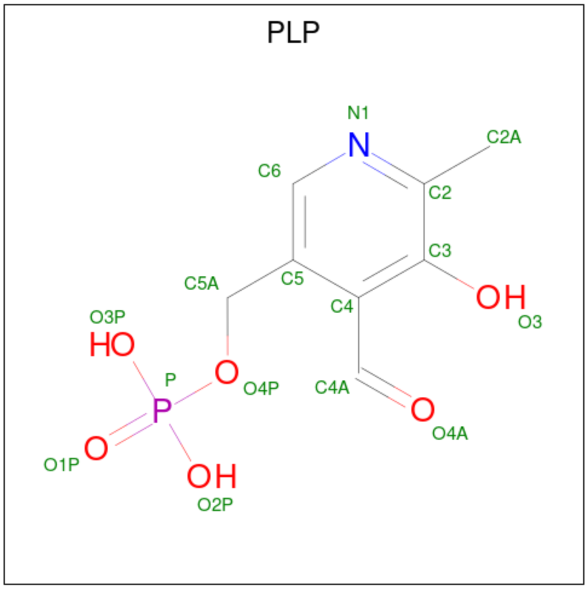

Mol	Chain	Residues	Atoms					ZeroOcc	AltConf
2	A	1	Total 15	C 8	N 1	O 5	P 1	0	0
2	B	1	Total 15	C 8	N 1	O 5	P 1	0	0

- Molecule 3 is 3-[4-(3-bromo-4-fluorobenzoyl)piperazin-1-yl]-7H-cyclopenta[c]pyridin-7-one (CCD ID: A1A2M) (formula: $C_{19}H_{15}BrFN_3O_2$) (labeled as "Ligand of Interest" by depositor).

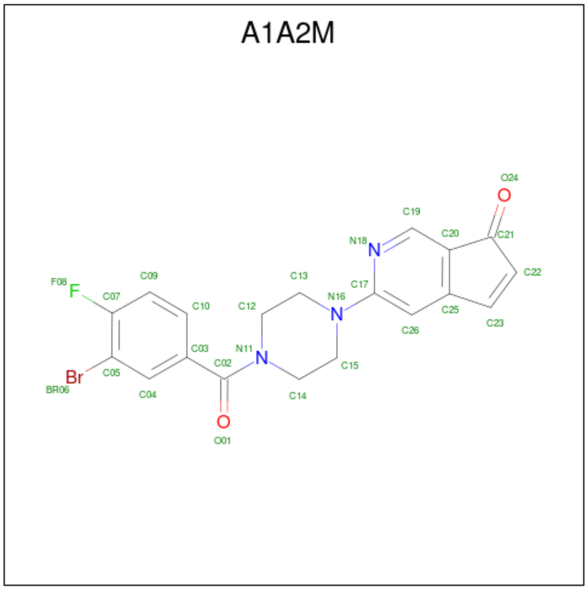

Mol	Chain	Residues	Atoms	ZeroOcc	AltConf
3	A	1	Total Br C F N O<br>26 1 19 1 3 2	0	0
3	B	1	Total Br C F N O<br>26 1 19 1 3 2	0	0

- Molecule 4 is water.

Mol	Chain	Residues	Atoms	ZeroOcc	AltConf
4	A	493	Total O<br>493 493	0	0
4	B	433	Total O<br>433 433	0	0

For Manuscript Review

3 Residue-property plots (i)

These plots are drawn for all protein, RNA, DNA and oligosaccharide chains in the entry. The first graphic for a chain summarises the proportions of the various outlier classes displayed in the second graphic. The second graphic shows the sequence view annotated by issues in geometry and electron density. Residues are color-coded according to the number of geometric quality criteria for which they contain at least one outlier: green = 0, yellow = 1, orange = 2 and red = 3 or more. A red dot above a residue indicates a poor fit to the electron density (RSRZ > 2). Stretches of 2 or more consecutive residues without any outlier are shown as a green connector. Residues present in the sample, but not in the model, are shown in grey.

• Molecule 1: Adenosylmethionine-8-amino-7-oxononanoate aminotransferase

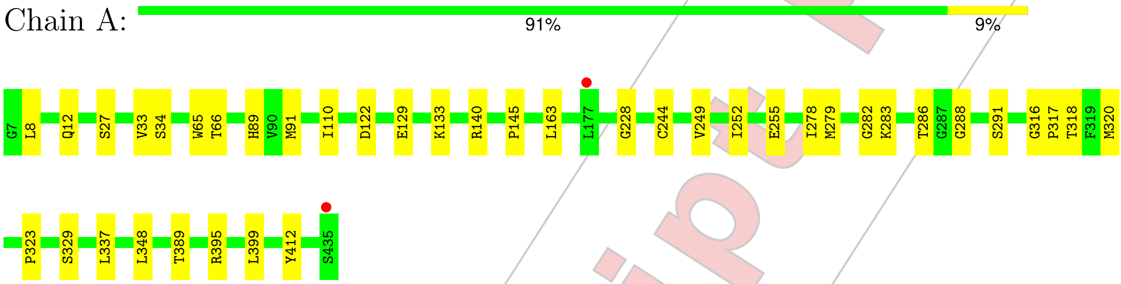

• Molecule 1: Adenosylmethionine-8-amino-7-oxononanoate aminotransferase

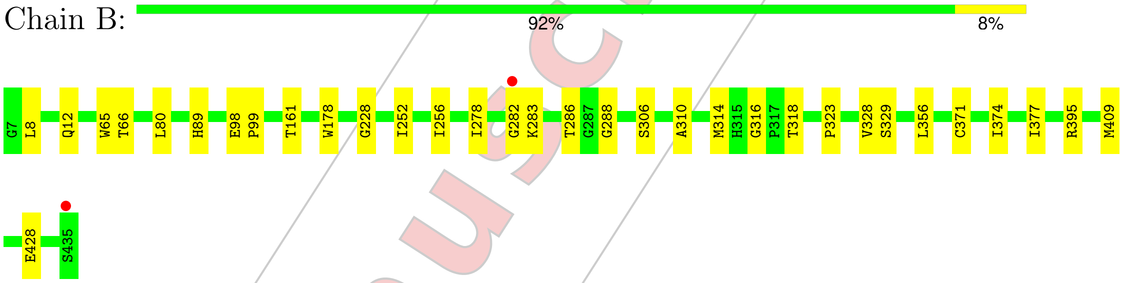

For Manuscript Review

4 Data and refinement statistics (i)

Property	Value	Source
Space group	P 21 21 21	Depositor
Cell constants<br>a, b, c, α, β, γ	63.14Å 66.29Å 204.84Å<br>90.00° 90.00° 90.00°	Depositor
Resolution (Å)	60.34 – 1.97<br>60.34 – 1.97	Depositor<br>EDS
% Data completeness<br>(in resolution range)	96.2 (60.34-1.97)<br>96.2 (60.34-1.97)	Depositor<br>EDS
R_{merge}	0.05	Depositor
R_{sym}	(Not available)	Depositor
$< I/\sigma(I) >$ [1]	2.23 (at 1.97Å)	Xtriage
Refinement program	PHENIX (1.18.2_3874)	Depositor
R, R_{free}	0.154 , 0.193<br>0.154 , 0.193	Depositor<br>DCC
R_{free} test set	1846 reflections (2.99%)	wwPDB-VP
Wilson B-factor (Å^2)	20.4	Xtriage
Anisotropy	0.079	Xtriage
Bulk solvent k_{sol}(e/Å^3), B_{sol}(Å^2)	0.36 , 51.1	EDS
L-test for twinning[2]	$< \|L\| > = 0.49$, $< L^2 > = 0.32$	Xtriage
Estimated twinning fraction	0.023 for k,h,-l	Xtriage
F_o,F_c correlation	0.96	EDS
Total number of atoms	7480	wwPDB-VP
Average B, all atoms (Å^2)	21.0	wwPDB-VP

Xtriage's analysis on translational NCS is as follows: *The largest off-origin peak in the Patterson function is 4.83% of the height of the origin peak. No significant pseudotranslation is detected.*

[1]Intensities estimated from amplitudes.

[2]Theoretical values of $< |L| >$, $< L^2 >$ for acentric reflections are 0.5, 0.333 respectively for untwinned datasets, and 0.375, 0.2 for perfectly twinned datasets.

For Manuscript Review

5 Model quality (i)

5.1 Standard geometry (i)

Bond lengths and bond angles in the following residue types are not validated in this section: A1A2M, PLP

The Z score for a bond length (or angle) is the number of standard deviations the observed value is removed from the expected value. A bond length (or angle) with $|Z| > 5$ is considered an outlier worth inspection. RMSZ is the root-mean-square of all Z scores of the bond lengths (or angles).

Mol	Chain	Bond lengths		Bond angles	
		RMSZ	#$|Z|$ >5	RMSZ	#$|Z|$ >5
1	A	0.26	0/3314	0.50	0/4532
1	B	0.24	0/3327	0.48	0/4546
All	All	0.25	0/6641	0.49	0/9078

There are no bond length outliers.

There are no bond angle outliers.

There are no chirality outliers.

There are no planarity outliers.

5.2 Too-close contacts (i)

In the following table, the Non-H and H(model) columns list the number of non-hydrogen atoms and hydrogen atoms in the chain respectively. The H(added) column lists the number of hydrogen atoms added and optimized by MolProbity. The Clashes column lists the number of clashes within the asymmetric unit, whereas Symm-Clashes lists symmetry-related clashes.

Mol	Chain	Non-H	H(model)	H(added)	Clashes	Symm-Clashes
1	A	3228	0	3205	23	0
1	B	3244	0	3218	20	0
2	A	15	0	6	0	0
2	B	15	0	6	1	0
3	A	26	0	0	0	0
3	B	26	0	0	1	0
4	A	493	0	0	0	0
4	B	433	0	0	1	0
All	All	7480	0	6435	38	0

The all-atom clashscore is defined as the number of clashes found per 1000 atoms (including hydrogen atoms). The all-atom clashscore for this structure is 3.

For Manuscript Review

All (38) close contacts within the same asymmetric unit are listed below, sorted by their clash magnitude.

Atom-1	Atom-2	Interatomic distance (Å)	Clash overlap (Å)
1:A:255:GLU:HG3	1:A:279:MET:HE3	1.72	0.69
1:B:252:ILE:HG12	1:B:278:ILE:HB	1.77	0.65
1:A:91:MET:HB2	3:B:502:A1A2M:O24	2.00	0.61
1:A:283:LYS:HE3	1:B:318:THR:OG1	2.01	0.60
1:A:286:THR:HG22	1:A:329[A]:SER:OG	2.05	0.57
1:B:395:ARG:CZ	1:B:428:GLU:HG2	2.36	0.56
1:B:286:THR:HG22	1:B:329[A]:SER:OG	2.05	0.56
1:A:318:THR:OG1	1:B:283:LYS:HE3	2.08	0.54
1:B:8:LEU:HD22	1:B:12:GLN:HB3	1.89	0.54
1:B:356:LEU:HD22	1:B:377:ILE:HD11	1.90	0.52
1:A:348:LEU:HD11	1:A:412:TYR:HA	1.91	0.52
1:A:8:LEU:HD22	1:A:12:GLN:HB3	1.93	0.50
1:A:89:HIS:HA	1:A:323:PRO:HD2	1.94	0.50
1:A:122:ASP:O	1:A:291:SER:OG	2.23	0.49
1:B:89:HIS:HA	1:B:323:PRO:HD2	1.95	0.49
1:B:80:LEU:HB2	1:B:328[B]:VAL:CG2	2.44	0.47
1:A:389:THR:HG22	1:A:399:LEU:O	2.15	0.47
1:A:129:GLU:OE2	1:A:163[B]:LEU:HB3	2.16	0.46
1:B:65:TRP:CD1	1:B:65:TRP:N	2.83	0.46
1:A:133:LYS:HG3	1:B:178:TRP:CH2	2.52	0.45
1:A:395:ARG:HH21	1:A:395:ARG:HG3	1.81	0.45
1:B:371:CYS:SG	4:B:1017:HOH:O	2.43	0.45
1:A:244:CYS:HB3	1:A:249:VAL:O	2.18	0.44
1:B:256:ILE:HG22	1:B:283:LYS:HG3	1.99	0.44
1:B:66:THR:HB	1:B:288:GLY:HA2	2.01	0.43
1:A:65:TRP:CD1	1:A:65:TRP:N	2.84	0.43
1:A:129:GLU:HG3	1:B:161:THR:HG22	2.00	0.43
1:A:66:THR:HB	1:A:288:GLY:HA2	2.00	0.43
1:B:283:LYS:NZ	2:B:501:PLP:O3	2.52	0.43
1:A:252:ILE:HG12	1:A:278:ILE:HB	2.00	0.42
1:B:310:ALA:HB1	1:B:314[A]:MET:HE3	2.01	0.42
1:B:98:GLU:HB3	1:B:99:PRO:HD3	2.01	0.42
1:A:27:SER:HB2	1:B:306:SER:HB3	2.02	0.42
1:B:374:ILE:HG12	1:B:409:MET:HG2	2.02	0.42
1:A:110:ILE:HG21	1:A:337:LEU:HD11	2.00	0.41
1:A:317:PRO:HD2	1:A:320:MET:HB2	2.02	0.41
1:A:33:VAL:HG12	1:A:34[B]:SER:O	2.22	0.40
1:A:140:ARG:HG2	1:A:145:PRO:HB3	2.04	0.40

There are no symmetry-related clashes.

For Manuscript Review

5.3 Torsion angles (i)

5.3.1 Protein backbone (i)

In the following table, the Percentiles column shows the percent Ramachandran outliers of the chain as a percentile score with respect to all X-ray entries followed by that with respect to entries of similar resolution.

The Analysed column shows the number of residues for which the backbone conformation was analysed, and the total number of residues.

Mol	Chain	Analysed	Favoured	Allowed	Outliers	Percentiles	
1	A	432/429 (101%)	421 (98%)	8 (2%)	3 (1%)	19	10
1	B	433/429 (101%)	421 (97%)	9 (2%)	3 (1%)	19	10
All	All	865/858 (101%)	842 (97%)	17 (2%)	6 (1%)	19	10

All (6) Ramachandran outliers are listed below:

Mol	Chain	Res	Type
1	B	282	GLY
1	A	282	GLY
1	A	228	GLY
1	B	228	GLY
1	A	316	GLY
1	B	316	GLY

5.3.2 Protein sidechains (i)

In the following table, the Percentiles column shows the percent sidechain outliers of the chain as a percentile score with respect to all X-ray entries followed by that with respect to entries of similar resolution.

The Analysed column shows the number of residues for which the sidechain conformation was analysed, and the total number of residues.

Mol	Chain	Analysed	Rotameric	Outliers	Percentiles	
1	A	328/325 (101%)	328 (100%)	0	100	100
1	B	329/325 (101%)	329 (100%)	0	100	100
All	All	657/650 (101%)	657 (100%)	0	100	100

There are no protein residues with a non-rotameric sidechain to report.

Sometimes sidechains can be flipped to improve hydrogen bonding and reduce clashes. All (1) such sidechains are listed below:

For Manuscript Review

Mol	Chain	Res	Type
1	B	42	HIS

5.3.3 RNA (i)

There are no RNA molecules in this entry.

5.4 Non-standard residues in protein, DNA, RNA chains (i)

There are no non-standard protein/DNA/RNA residues in this entry.

5.5 Carbohydrates (i)

There are no oligosaccharides in this entry.

5.6 Ligand geometry (i)

4 ligands are modelled in this entry.

In the following table, the Counts columns list the number of bonds (or angles) for which Mogul statistics could be retrieved, the number of bonds (or angles) that are observed in the model and the number of bonds (or angles) that are defined in the Chemical Component Dictionary. The Link column lists molecule types, if any, to which the group is linked. The Z score for a bond length (or angle) is the number of standard deviations the observed value is removed from the expected value. A bond length (or angle) with $|Z| > 2$ is considered an outlier worth inspection. RMSZ is the root-mean-square of all Z scores of the bond lengths (or angles).

Mol	Type	Chain	Res	Link	Bond lengths			Bond angles		
					Counts	RMSZ	$\#\|Z\| > 2$	Counts	RMSZ	$\#\|Z\| > 2$
2	PLP	B	501	1	15,15,16	0.88	1 (6%)	21,22,23	1.45	2 (9%)
2	PLP	A	501	1	15,15,16	0.82	1 (6%)	21,22,23	1.13	2 (9%)
3	A1A2M	A	502	-	29,29,29	2.26	12 (41%)	38,42,42	1.98	13 (34%)
3	A1A2M	B	502	-	29,29,29	2.31	12 (41%)	38,42,42	1.89	13 (34%)

In the following table, the Chirals column lists the number of chiral outliers, the number of chiral centers analysed, the number of these observed in the model and the number defined in the Chemical Component Dictionary. Similar counts are reported in the Torsion and Rings columns. '-' means no outliers of that kind were identified.

Mol	Type	Chain	Res	Link	Chirals	Torsions	Rings
2	PLP	B	501	1	-	0/6/6/8	0/1/1/1

Continued on next page...

For Manuscript Review

Continued from previous page...

Mol	Type	Chain	Res	Link	Chirals	Torsions	Rings
2	PLP	A	501	1	-	0/6/6/8	0/1/1/1
3	A1A2M	A	502	-	-	0/12/31/31	0/4/4/4
3	A1A2M	B	502	-	-	0/12/31/31	0/4/4/4

All (26) bond length outliers are listed below:

Mol	Chain	Res	Type	Atoms	Z	Observed(Å)	Ideal(Å)
3	B	502	A1A2M	C22-C23	4.88	1.57	1.36
3	A	502	A1A2M	C25-C23	4.59	1.57	1.43
3	A	502	A1A2M	C22-C23	4.48	1.55	1.36
3	B	502	A1A2M	C25-C23	4.43	1.56	1.43
3	B	502	A1A2M	C13-N16	4.15	1.53	1.46
3	A	502	A1A2M	C13-N16	4.13	1.53	1.46
3	A	502	A1A2M	C15-N16	3.46	1.52	1.46
3	B	502	A1A2M	C22-C21	3.34	1.55	1.46
3	A	502	A1A2M	C17-N16	3.20	1.44	1.37
3	B	502	A1A2M	C15-N16	3.15	1.52	1.46
3	B	502	A1A2M	C26-C17	3.06	1.44	1.39
3	B	502	A1A2M	C10-C03	3.03	1.44	1.39
3	B	502	A1A2M	C17-N16	3.02	1.44	1.37
3	A	502	A1A2M	C26-C17	2.96	1.44	1.39
3	A	502	A1A2M	C22-C21	2.94	1.54	1.46
3	B	502	A1A2M	C02-N11	2.86	1.40	1.34
3	A	502	A1A2M	C02-N11	2.84	1.40	1.34
3	A	502	A1A2M	C10-C03	2.53	1.43	1.39
3	B	502	A1A2M	C03-C02	2.43	1.54	1.50
3	A	502	A1A2M	C03-C02	2.40	1.54	1.50
2	B	501	PLP	C4A-C4	-2.34	1.46	1.51
3	B	502	A1A2M	C09-C07	2.29	1.42	1.37
2	A	501	PLP	C4A-C4	-2.22	1.47	1.51
3	A	502	A1A2M	F08-C07	-2.22	1.29	1.35
3	B	502	A1A2M	F08-C07	-2.20	1.29	1.35
3	A	502	A1A2M	C09-C07	2.03	1.42	1.37

All (30) bond angle outliers are listed below:

Mol	Chain	Res	Type	Atoms	Z	Observed(^{o})	Ideal(^{o})
2	B	501	PLP	O4P-C5A-C5	4.53	117.85	109.36
3	A	502	A1A2M	C09-C07-C05	-4.18	115.85	121.45
3	A	502	A1A2M	C12-N11-C14	-4.04	104.45	112.68
3	B	502	A1A2M	C09-C07-C05	-3.99	116.11	121.45
3	B	502	A1A2M	C12-N11-C14	-3.81	104.91	112.68

Continued on next page...

For Manuscript Review

Continued from previous page...

Mol	Chain	Res	Type	Atoms	Z	Observed(°)	Ideal(°)
3	A	502	A1A2M	C10-C03-C04	-3.67	115.00	119.25
2	B	501	PLP	C4A-C4-C5	3.60	124.65	120.94
2	A	501	PLP	O4P-C5A-C5	3.53	115.98	109.36
3	B	502	A1A2M	C03-C04-C05	3.49	123.62	119.79
3	B	502	A1A2M	C10-C03-C04	-3.48	115.23	119.25
3	A	502	A1A2M	C25-C26-C17	3.22	119.39	117.11
3	A	502	A1A2M	C03-C04-C05	3.05	123.14	119.79
3	B	502	A1A2M	C20-C21-C22	2.75	109.43	106.04
3	A	502	A1A2M	C15-C14-N11	2.71	115.80	110.42
3	B	502	A1A2M	C25-C26-C17	2.70	119.03	117.11
2	A	501	PLP	C4A-C4-C5	2.57	123.58	120.94
3	B	502	A1A2M	C15-C14-N11	2.51	115.42	110.42
3	A	502	A1A2M	C12-C13-N16	2.43	115.90	110.78
3	A	502	A1A2M	C04-C05-C07	2.42	122.88	119.19
3	A	502	A1A2M	O24-C21-C22	-2.35	123.13	126.74
3	A	502	A1A2M	C20-C21-C22	2.32	108.90	106.04
3	A	502	A1A2M	N18-C17-N16	2.28	120.03	116.76
3	A	502	A1A2M	C12-N11-C02	2.27	129.83	122.79
3	B	502	A1A2M	N18-C17-N16	2.26	119.99	116.76
3	B	502	A1A2M	C04-C05-C07	2.24	122.61	119.19
3	B	502	A1A2M	C19-N18-C17	2.21	121.02	117.41
3	B	502	A1A2M	C12-N11-C02	2.20	129.59	122.79
3	B	502	A1A2M	C12-C13-N16	2.19	115.39	110.78
3	A	502	A1A2M	C19-N18-C17	2.14	120.91	117.41
3	B	502	A1A2M	O24-C21-C22	-2.03	123.62	126.74

There are no chirality outliers.

There are no torsion outliers.

There are no ring outliers.

2 monomers are involved in 2 short contacts:

Mol	Chain	Res	Type	Clashes	Symm-Clashes
2	B	501	PLP	1	0
3	B	502	A1A2M	1	0

The following is a two-dimensional graphical depiction of Mogul quality analysis of bond lengths, bond angles, torsion angles, and ring geometry for all instances of the Ligand of Interest. In addition, ligands with molecular weight > 250 and outliers as shown on the validation Tables will also be included. For torsion angles, if less then 5% of the Mogul distribution of torsion angles is within 10 degrees of the torsion angle in question, then that torsion angle is considered an outlier. Any bond that is central to one or more torsion angles identified as an outlier by Mogul will be

For Manuscript Review

highlighted in the graph. For rings, the root-mean-square deviation (RMSD) between the ring in question and similar rings identified by Mogul is calculated over all ring torsion angles. If the average RMSD is greater than 60 degrees and the minimal RMSD between the ring in question and any Mogul-identified rings is also greater than 60 degrees, then that ring is considered an outlier. The outliers are highlighted in purple. The color gray indicates Mogul did not find sufficient equivalents in the CSD to analyse the geometry.

Ligand A1A2M A 502

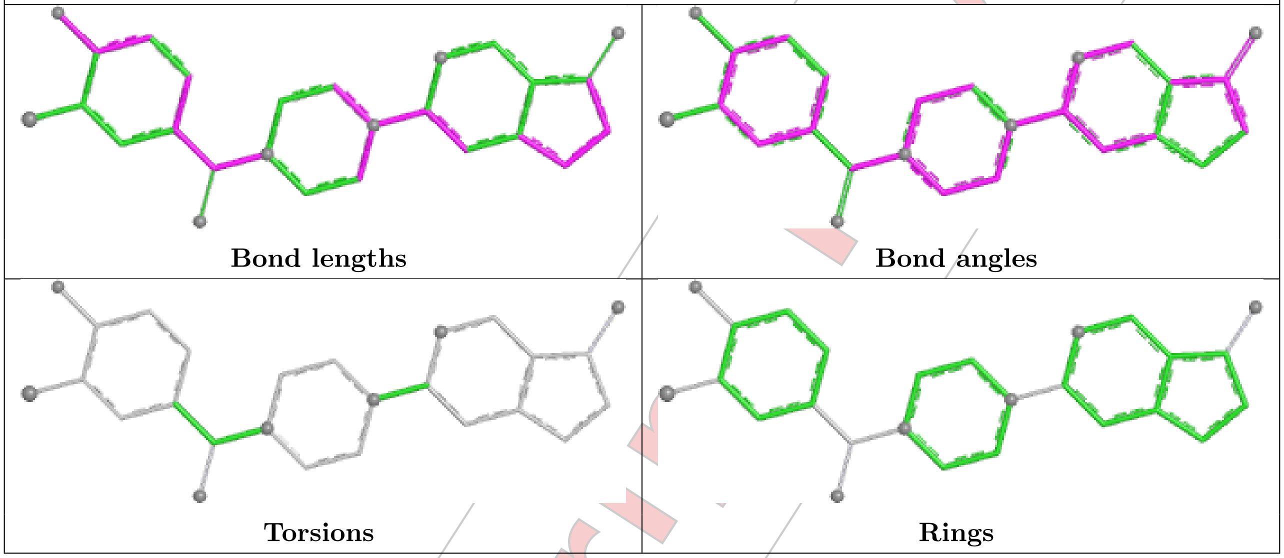

Ligand A1A2M B 502

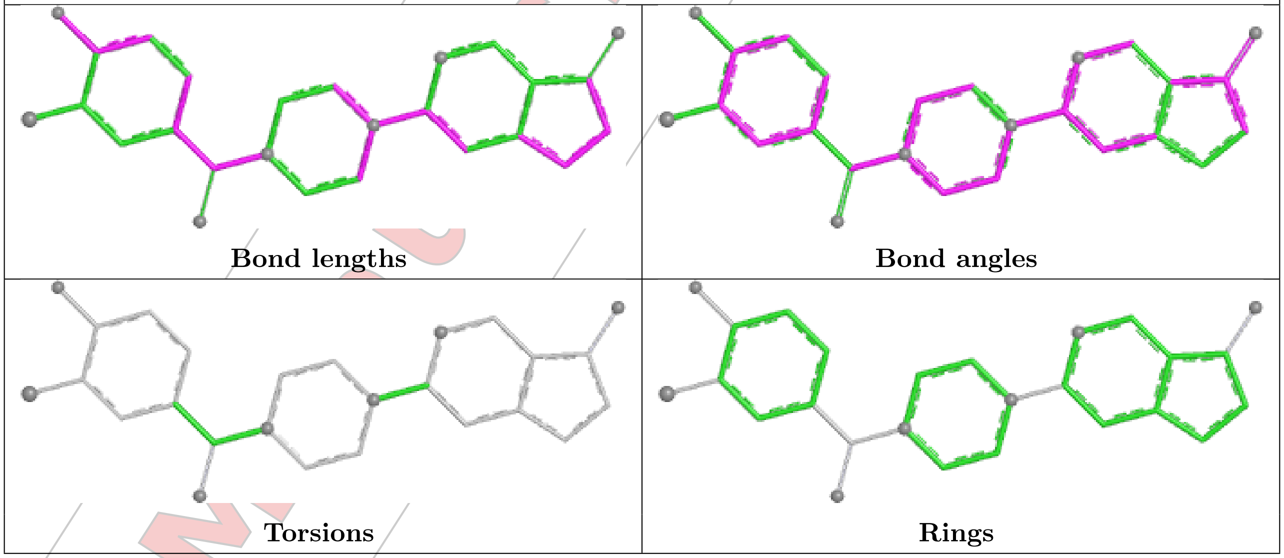

5.7 Other polymers (i)

There are no such residues in this entry.

For Manuscript Review

5.8 Polymer linkage issues (i)

There are no chain breaks in this entry.

For Manuscript Review

6 Fit of model and data (i)

6.1 Protein, DNA and RNA chains (i)

In the following table, the column labelled '#RSRZ> 2' contains the number (and percentage) of RSRZ outliers, followed by percent RSRZ outliers for the chain as percentile scores relative to all X-ray entries and entries of similar resolution. The OWAB column contains the minimum, median, 95th percentile and maximum values of the occupancy-weighted average B-factor per residue. The column labelled 'Q< 0.9' lists the number of (and percentage) of residues with an average occupancy less than 0.9.

Mol	Chain	Analysed	<RSRZ>	#RSRZ>2			OWAB(Å^2)	Q<0.9
1	A	429/429 (100%)	-0.53	2 (0%)	87	91	7, 18, 30, 50	5 (1%)
1	B	429/429 (100%)	-0.50	2 (0%)	87	91	7, 20, 33, 50	6 (1%)
All	All	858/858 (100%)	-0.52	4 (0%)	87	91	7, 19, 32, 50	11 (1%)

All (4) RSRZ outliers are listed below:

Mol	Chain	Res	Type	RSRZ
1	A	435	SER	3.4
1	B	435	SER	3.2
1	A	177	LEU	3.0
1	B	282	GLY	2.0

6.2 Non-standard residues in protein, DNA, RNA chains (i)

There are no non-standard protein/DNA/RNA residues in this entry.

6.3 Carbohydrates (i)

There are no oligosaccharides in this entry.

6.4 Ligands (i)

In the following table, the Atoms column lists the number of modelled atoms in the group and the number defined in the chemical component dictionary. The B-factors column lists the minimum, median, 95th percentile and maximum values of B factors of atoms in the group. The column labelled 'Q< 0.9' lists the number of atoms with occupancy less than 0.9.

Mol	Type	Chain	Res	Atoms	RSCC	RSR	B-factors(Å^2)	Q<0.9
2	PLP	B	501	15/16	0.96	0.06	11,14,17,17	0

Continued on next page...

For Manuscript Review

Continued from previous page...

Mol	Type	Chain	Res	Atoms	RSCC	RSR	B-factors($Å^2$)	Q<0.9
2	PLP	A	501	15/16	0.97	0.06	10,12,16,16	0
3	A1A2M	A	502	26/26	0.99	0.06	11,17,25,25	0
3	A1A2M	B	502	26/26	0.99	0.05	12,18,23,27	0

The following is a graphical depiction of the model fit to experimental electron density of all instances of the Ligand of Interest. In addition, ligands with molecular weight > 250 and outliers as shown on the geometry validation Tables will also be included. Each fit is shown from different orientation to approximate a three-dimensional view.

Electron density around A1A2M A 502:

$2mF_o$-DF_c (at 0.7 rmsd) in gray
mF_o-DF_c (at 3 rmsd) in purple (negative)
and green (positive)

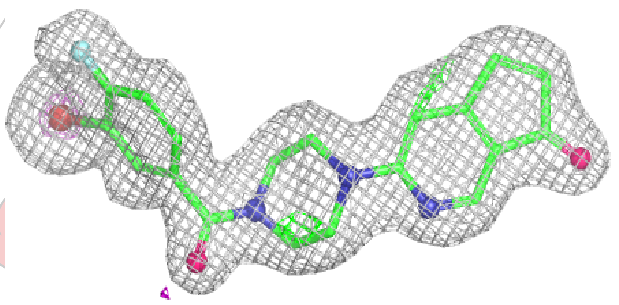

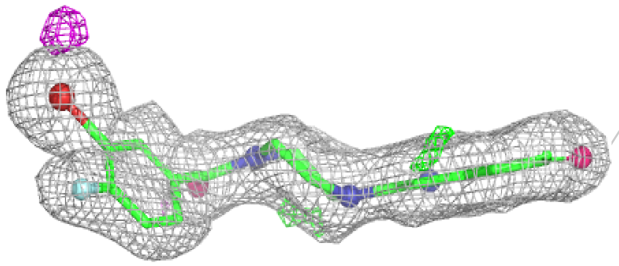

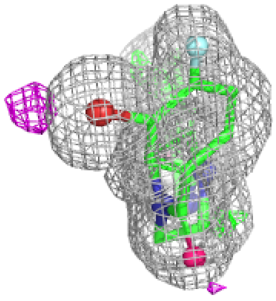

For Manuscript Review

Electron density around A1A2M B 502:

$2mF_o$-DF_c (at 0.7 rmsd) in gray
mF_o-DF_c (at 3 rmsd) in purple (negative)
and green (positive)

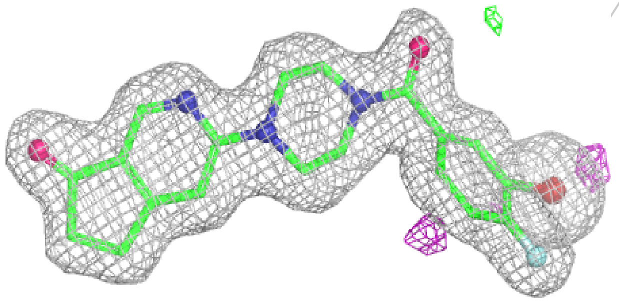

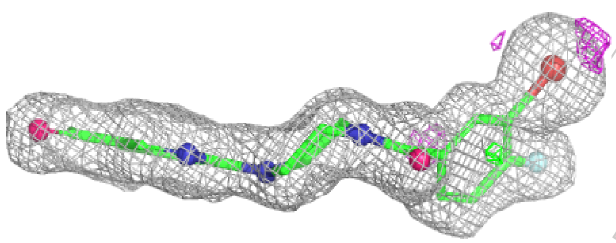

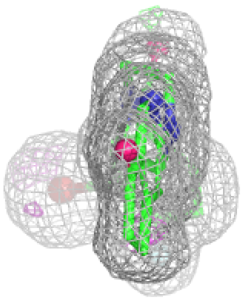

6.5 Other polymers (i)

There are no such residues in this entry.

For Manuscript Review
